# Terpyridine-Functionalized Calixarenes: Synthesis, Characterization and Anion Sensing Applications

**DOI:** 10.3390/molecules26010087

**Published:** 2020-12-27

**Authors:** Nicola Y. Edwards, David M. Schnable, Ioana R. Gearba-Dolocan, Jenna L. Strubhar

**Affiliations:** 1 Department of Chemistry and Biochemistry, Misericordia University, Dallas, PA 18612, USA; dschnable@gmail.com (D.M.S.); jennastrubhar@gmail.com (J.L.S.); 2 Department of Chemistry, The University of Texas at Austin, Austin, TX 78712, USA; gearba@austin.utexas.edu

**Keywords:** calix[4]arene, anion, sensors, lanthanides, fluorescence studies, UV–vis studies, quantum yields, lifetime measurements

## Abstract

Lanthanide complexes have been developed and are reported herein. These complexes were derived from a terpyridine-functionalized calix[4]arene ligand, chelated with Tb^3+^ and Eu^3+^. Synthesis of these complexes was achieved in two steps from a calix[4]arene derivative: (1) amide coupling of a calix[4]arene bearing carboxylic acid functionalities and (2) metallation with a lanthanide triflate salt. The ligand and its complexes were characterized by NMR (^1^H and ^13^C), fluorescence and UV-vis spectroscopy as well as MS. The photophysical properties of these complexes were studied; high molar absorptivity values, modest quantum yields and luminescence lifetimes on the ms timescale were obtained. Anion binding results in a change in the photophysical properties of the complexes. The anion sensing ability of the Tb(III) complex was evaluated via visual detection, UV-vis and fluorescence studies. The sensor was found to be responsive towards a variety of anions, and large binding constants were obtained for the coordination of anions to the sensor.

## 1. Introduction

### 1.1. Lanthanide Complexes as Anion Sensors

Developing sensors for the detection and quantification of anions is a major area of interest in supramolecular chemistry. This is due to the pivotal roles that these sensors play in many arenas including environmental, biological and medicinal arenas [1,2,3,4,5,6]. Lanthanide-based complexes have been developed and used extensively as sensors for a variety of analytes; many of these sensors are now used in commercial settings [7,8,9,10]. These complexes are standouts in this arena; anion sensing is no exception to this [11,12,13]. For a lanthanide complex—or any compound—to act as an anion sensor, two criteria must be met: (1) There must be binding of the sensor to anion, and (2) The sensor must display a readout (color, luminescence, etc.), and this readout must be modulated upon anion binding. Anions readily coordinate to lanthanide centers due to their potent Lewis acidity [14,15,16]. Lanthanide complexes boast excellent photophysical properties such as relatively long-lived luminescence (on the ms timescale). Furthermore, there are changes in the photophysical properties of these complexes that occur upon anion coordination which can be harnessed in a sensing scheme.

The terpyridine moiety is an effective sensitizer of lanthanide luminescence, and hence this moiety has been incorporated into several lanthanide-based anion sensors [17,18,19]; none of these sensors utilize the calixarene scaffold [20,21,22,23]. The calixarene scaffold has been frequently used in the construction of anion (and other) sensors as it imparts a high degree of pre-organization of the recognition groups leading to enhanced binding affinities of target molecules or ions [24,25]. The current work details the development of calixarene-based sensors for anions that have been synthesized in two steps from a calixarene derivative. A calix[4]arene scaffold with carboxylic acid functionalities at the lower rim was coupled to an amine derivative bearing a terpyridine moiety. The resulting compound was metallated by stirring it overnight with the appropriate lanthanide(III) triflate salt to form either the Eu(III) or Tb(III) complex. These lanthanide complexes have large molar absorptivity values, long luminescent lifetimes and modest quantum yields. Furthermore, these complexes were shown by visual detection, fluorescence and UV-vis studies to sense a variety of anions.

### 1.2. Sensor Design

Parker, Gunnlaugsson and others have pioneered the development of anion sensors based on antenna-sensitized lanthanide luminescence [26,27,28,29,30]. Two sensing paradigms that were developed and used for the basis of our study are described as follows: (1) Coordinatively-unsaturated metal complexes are used, in which anion binding leads to a change in the photophysical properties of the metal complex, due to the displacement of solvent molecules from the metal center. (2) The sensitizer is designed so that it is equipped with anion binding moieties; anion coordination leads to a change in the readout of the luminescence of the sensor due to changes that occur to the sensitizer upon anion binding [14].

**Scheme 1:**
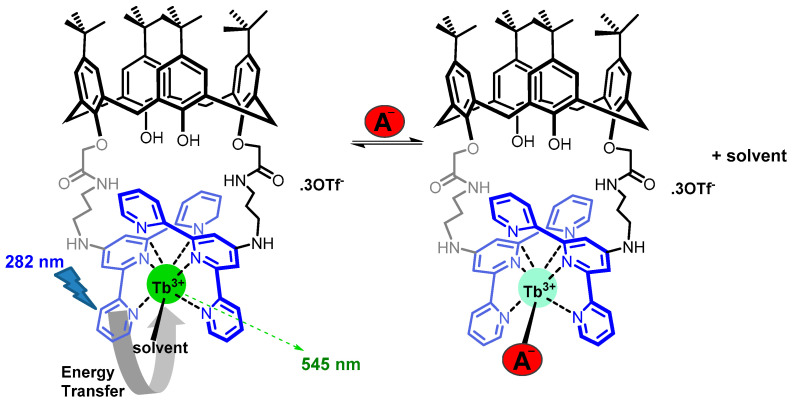
Sensor Design of [Tb.**1**]^3+^: Antenna-sensitized lanthanide luminescence is effected when the terpyridine moiety is excited with UV light. There is a change in the photophysical properties of the metal center upon binding due to (**a**) displacement of the solvent molecules with the anion and/or (**b**) anion binding to the linkers (through amide and amine moieties) of the calix[4]arene.

Anion receptors that were constructed using the calix[4]arene scaffold, containing linkers with amide functionalities and pyridine moieties, were previously reported by our group [31]. We showed by ^1^H NMR studies that these receptors were responsive to carboxylates and H_2_PO_4_^−^ with binding constants that ranged from 14 to 275 M^−1^ (in DMSO). Building on this earlier model system, we set our sights on developing sensors in which the linkers appended from the calix[4]arene scaffold terminated in terpyridine moieties. These terpyridine serve the dual purpose of binding lanthanide salts and acting as the sensitizing antenna (Scheme 1).

In our sensor design, we postulated that binding of the anions would change the photophysical properties of the complex due to: (1) The coordinatively unsaturated nature of the complexes; anions should bind directly to the metal center leading to displacement of solvent molecules and/or ligands. (2) The linkers of the metal complexes themselves are rich in anion binding moieties (specifically amide and amine moieties); binding to such moieties (via hydrogen bonding) is predicted to change the sensitizing ability of the ligand [32,33]. Either or both changes could form the basis of an appropriate signaling mechanism for our system. This sensor is also ripe with opportunities for diversification (via the antenna, metal center and linkers) for future pattern-based sensing applications [34]. In the following paragraphs, an investigation of this sensor is presented. The investigation begins with the synthesis and characterization of the complexes and is followed by the metal binding and photophysical studies. Finally, insights into the capabilities of the sensor are detailed.

## 2. Results and Discussion

### 2.1. Synthesis and Characterization of Ligand and its Lanthanide-Based Complexes

**Scheme 2:**
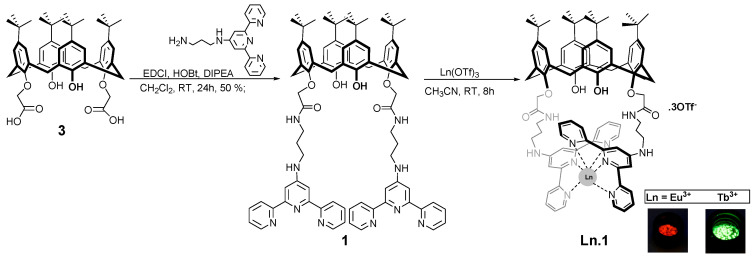
Synthesis of ligand **1** and lanthanide-based complexes.

With sensing applications in mind, it was necessary that the synthetic route be facile, amenable to scale-up and with the potential to create a library of ligands from available precursor compounds; amide coupling was chosen. The synthetic route to ligand **1** is shown in Scheme 2 and is summarized as follows. Ligand **1** was obtained by coupling the calix[4]arene dicarboxylic acid **3** [22] and N-[2,2′;6′,2″]terpyridin-4′-yl-propane-1,3-diamine [35] using *N*-Ethyl-*N*′-(3-dimethylaminopropyl)carbodiimide hydrochloride (EDCI), 1-Hydroxybenzotriazole hydrate (HOBt) in the presence of *N*,*N*-Diisopropylethylamine (DIPEA) in CH_2_Cl_2_. Subsequent work-up and purification via column-chromatography afforded **1** in 48% percent yield. The structure of ligand **1** was confirmed using ^1^H and ^13^C NMR spectroscopy and MS. Key resonances in the ^1^H NMR spectrum were found in the region of 7–9 ppm due to the terpyridine moieties and the aromatic moieties of the calixarene scaffold. There were also singlets at 4.65 ppm due to the methylene linker as well as at 1.09 and 1.06 ppm due to the *tert*-butyl groups. Of particular importance are the doublet of doublets found at 4.18 and 3.30 ppm due to the methylene bridges indicating that ligand **1** has C_2*v*_ symmetry and is locked in the cone conformation; this was also supported by ^13^C NMR data that showed a set of overlapping resonances at 31.3 ppm. The presence of a peak due to the doubly-charged ion [M+2H]^2+^ in the HRMS also supported formation of ligand **1.** Metallation of ligand **1** with a lanthanide(III) triflate salt was effected by stirring equimolar quantities of the salt and the ligand overnight at room temperature in CH_3_CN. Precipitation via hexanes of a concentrated solution of **1** in CH_2_Cl_2_ was used to obtain the lanthanide-based complexes of **1.** The Eu(III) and Tb(III) complexes were isolated in 53% and 46% yield, respectively.

### 2.2. How Well Does Ligand **1** Bind Lanthanide(III) Ions?

The investigation began with ligand **1** as it was our entry point to the sensors and because of its own potential in future sensing applications. Specifically, there was interest in determining how easily metal complexation could be monitored in situ and the strength of the binding of this ligand with lanthanides. We, thus, investigated the binding of ligand **1** to Tb(III) and Eu(III) using UV-vis and fluorescence spectroscopy. For these studies, solutions of ligand **1** (9.7 µM) were titrated with 0–7 equivalents of lanthanide ions (as triflate salts) in CH_3_CN.

**Figure 1:**
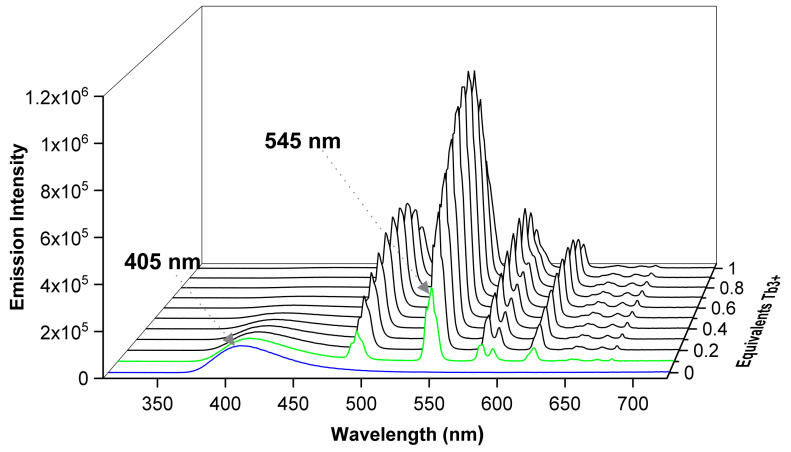
Fluorescence spectra representing the titration of ligand **1** (9.7 µM, acetonitrile) with 0 to 1 equivalents of Tb(OTf)_3_. Excitation at 280 nm; excitation and emission slit widths are 2 and 1 nm, respectively. Integration time = 0.2 s.

Fluorescence spectra representing the titration of ligand **1** with Tb(OTf)_3_ are shown in Figure 1. Prior to the addition of any equivalents of Tb(OTf)_3_, there is a peak centered at 410 nm due to the ligand. As the titration progresses, this peak decreases in intensity and is slightly red-shifted. There is the concomitant appearance of line spectra in the 450–700 nm region, signaling that the [Tb.**1**](OTf)_3_ had been formed in situ (see Section 2.3).

**Figure 2:**
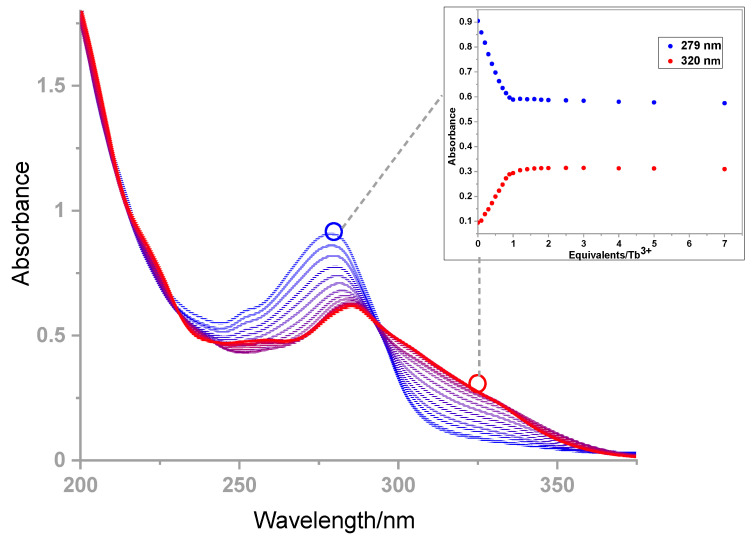
Absorption spectra resulting from the titration of ligand **1** (9.7 µM, acetonitrile) with 0 to 7 equivalents of Tb(OTf)_3_.

The same titration was repeated and monitored using UV–vis spectroscopy. Shown in Figure 2 is a series of absorption spectra resulting from the titration of ligand **1** with Tb(OTf)_3_. There is a decrease in the intensity of the peak at 279 nm (slightly red-shifted by ca. 6 nm) and the growth of a shoulder at 325 nm, indicating the complexation of the terpyridine moieties with the metal center. The absorption spectrum at the end of the titration matched that of the Tb.**1**(OTf)_3_ (see Section 2.3). Binding isotherms obtaining by plotting absorbance vs. Tb(OTf)_3_ concentration at 279 and 320 nm revealed the presence of an inflection point at 1 equivalent Tb(OTf)_3_ added, indicated 1:1 ligand: metal binding stoichiometry. These isotherms were subsequently analyzed using a 1:1 ligand: metal model in the nonlinear regression curve-fitting program Hypspec [36] and yielded formation constants of log *K* = 7.3 (±0.2) for Tb(III) and log *K* = 6.6 (±0.1) for Eu(III), respectively (see supplementary information). These binding constants are within the range of other Eu(III) and Tb(III) complexes based on terpyridine and other pyridine-based ligands [37,38]. For example, the log *K* for a 1:1 Eu(III) complex based on a tripodal terpyridine ligand was determined to be 7.2 (±0.3) in methanol [35]. The log *K* of a 1:1 Eu(III) complex based on a bis-bipyridinehenylphosphine oxide ligand was determined to be 5.8 (±0.5) in acetonitrile [39].

### 2.3. Photophysical Studies of Ligand **1** and Complexes

**Figure 3:**
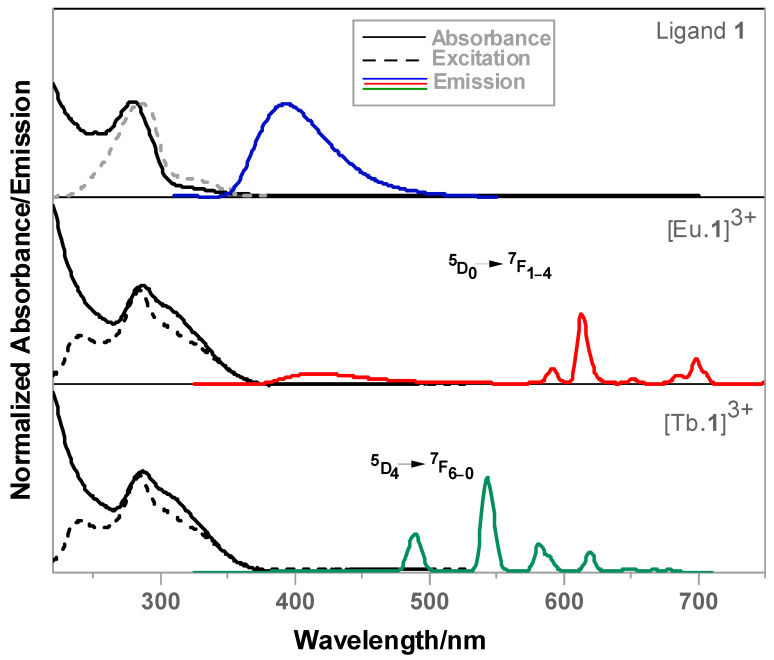
Normalized absorbance, excitation and fluorescence spectra of [Tb.**1**](OTf)_3_ (9.9 µM, acetonitrile) shown alongside the normalized ligand absorbance. Fluorescence spectrum conditions: emission: excitation at 280 nm; excitation: emission wavelengths: 394 (ligand), 543 (Tb) and 613 nm (Eu).

Next, the photophysical properties of ligand **1** and its complexes were examined; these data are summarized in Table 1. We begin our discussion of this section by firstly highlighting the absorption, excitation and emission spectra of ligand **1** and its complexes (Figure 3) and the information that was gleaned from these spectra. The spectra of the ligand and its Ln(III) Complexes (9.8 µM) were measured in CH_3_CN. The absorption spectrum of the ligand (top panel) is dominated by a peak centered at 282 nm (log ε = 4.70 in acetonitrile) due to the π → π* transition of the terpyridine moieties and the calixarene scaffold; a broad shoulder centered at ca. 296 nm is also present. The emission spectrum of the ligand resulting from excitation at 280 nm shows a single peak centered at 394 nm.

The dominant peaks in the absorption spectra of the lanthanide complexes (Figure 3—middle and bottom panels) show a 5 nm bathochromic shift with respect to ligand, indicating that the terpyridine moiety is responsible for complexation of the ligand; the shift is the result of a change in conformation from the *trans*, *trans* or *cis*, *trans* form to the *cis*, *cis* form [40]. We note that absorption for the ligand and complexes is considered to be a highly efficient process as indicated by the high values for molar absorptivity (logε: 4.17–4.70) that were obtained.

The emission spectra of the lanthanide complexes (Figure 3—middle and bottom panels), produced by exciting the complexes in the absorption band of the ligand, resulted in an intense metal-centered luminescence. This luminescence is displayed in the form of line spectra, ranging from 591 to 698 nm for Eu(III), and ranging from 488 to 677 nm for Tb(III). These line spectra serve as spectroscopic signatures of successful lanthanide complexation resulting from terpyridine-based ligand sensitization and hence energy transfer from ligand to metal. In the case of the Eu(III) complexes, energy is transferred from the ligand to the ^5^D_0_ excitation state of Eu(III), and there is subsequent deactivation to the ground state multiplets 7F_J_ (J = 0–4)). For the Tb(III) complexes, the ^5^D_4_ excited state and the ground state multiplets are 7F_J_ (J = 6–0). Similarities in the absorption and excitation spectra of the ligand and complexes are indicative of good “energy matches” between ligand and metal ion that resulted in this successful energy transfer. Blue (for ligand), green (for Tb) and red emission (for Eu) can be detected with the naked eye upon exposure of the ligand and complexes (in the solid state) to UV light (Scheme 2).

The complexes were then defined in terms of the requisite benchmarks of luminescence, i.e., the lifetime and quantum yield of the luminescence of the complexes. Lifetimes (Table 1) were obtained by measuring the luminescence decay at the maximum of the most dominant transitions (^5^D_4_ → ^7^F_5_ transition at 543 nm for Tb(III) and ^5^D_0_→ ^7^F_2_ transition at 613 nm for Eu(III)). In all cases, the decays were single exponential in nature indicating that the luminescence arose from one excited state. Rate constants were extracted from the single exponential fits and subsequently converted to lifetimes. For both lanthanide complexes, the lifetime measurements were ca. 1 ms—the typical timescale for lifetimes of lanthanide complexes.

The fluorescence quantum yields for the lanthanide complexes were measured in acetonitrile using the relative method [41,42]. In short, this method involves measuring the quantum yield of an analyte relative to a compound with a known fluorescence quantum yield. This is performed by recording the fluorescent emission spectrum of both compounds in solution, integrating this spectrum over the emission range and plotting this value against the absorbance of the solution at the excitation wavelength for multiple solutions at varying, but dilute, concentrations (A < 0.1). A linear trendline is fit to this plot, the gradient of which can be used to calculate the quantum yield of the analyte relative to the standard. This is performed according to the following equation:

$$\phi_{x}\;=\;\;\phi_{st}\left(\frac{grad_{x}}{grad_{st}}\right)\left(\frac{\eta_{x}^{2}}{\eta_{st}^{2}}\right)$$

*φ_x_*, *η_x_* and *grad_x_* represent the fluorescence quantum yield, refractive index and gradient of the linear trendline, respectively. The quantum yields were modest: 4.5% for the Tb(III) complex and 0.24% for the Eu(III) complex.

### 2.4. Anion Binding Studies—Assay Developemt

The Tb complex, [Tb.**1**](OTf)_3_, was the metal complex chosen to probe anion binding due to its higher quantum yield—a factor of 20 higher than its Eu counterpart. We selected anions (NO_3_^−^, H_2_PO_4_^−^, Cl^−^ and CH_3_CO_2_^−^-as tetrabutylammonium (TBA) salts) to determine if there were changes in the luminescence of [Tb.**1**](OTf)_3_. Luminescence changes were observed upon the addition of these anions when acetonitrile and methanol were used as solvents, but acetonitrile was ultimately chosen as it is easier to carry out studies over a longer period of time due to its slower rate of evaporation compared to methanol. We explored changes in the ground state of the complex (using absorption spectra) and its excited state (using fluorescence spectra). Two regions of the fluorescence spectrum were explored: (1) the π* to π transition centered at about 400 nm and (2) the Tb excited state to ground state transition (ca. 450–720 nm).

### 2.5. Binding Studies of the Tb.1(OTf)_3_ with Dihydrogen Phosphate

To determine the response of our sensor towards anions, [Tb.**1**](OTf)_3_ was titrated with solutions of anions as their TBA salts in CH_3_CN. We first determined if our sensor would exhibit a response towards triflate, the counter anion in our complex. Titration of up to 100 equivalents of TBA triflate did not change the overall response of the sensor by any appreciable amount.

**Figure 4:**
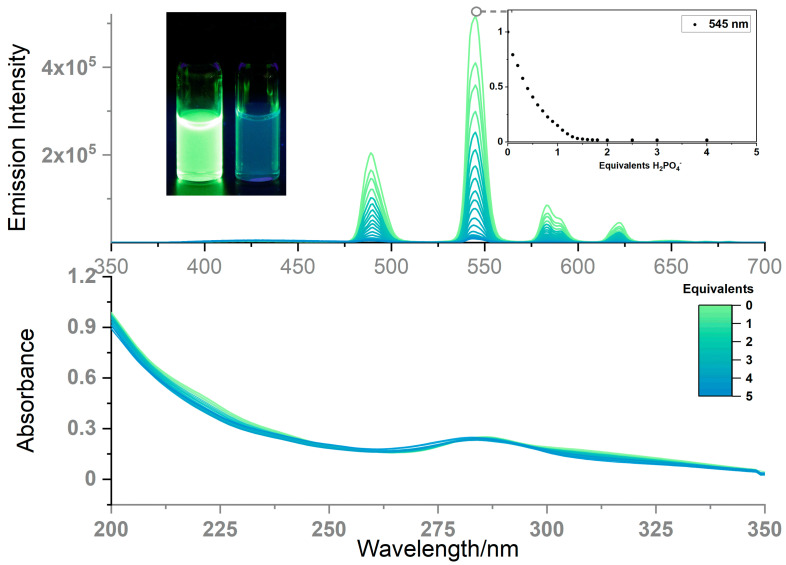
Top panel: 0.9 mM of [Tb.**1**](OTf)_3_ in the absence (left) and presence (right) of H_2_PO_4_^−^ (10 equivalents). Spectra resulting from the titration of [Tb.**1**](OTf)_3_ (9.9 µM, acetonitrile) with 0 to 7 equivalents of H_2_PO_4_^−^. Fluorescence (top panel) and UV-vis (bottom panel). The binding isotherm for the emission intensity at 545 nm in the fluorescence spectrum is shown as an inset in the top panel.

Shown in Figure 4 (top panel—left) is an example of the visual detection of an anion, H_2_PO_4_^−^, by [Tb.**1**](OTf)_3_. The complex changes from fluorescent green to blue in the presence of the anion indicating that the sensor exhibits a “turn-off” response towards H_2_PO_4_^−^. Fluorescence spectra (Figure 4—top panel) obtained upon the titration of H_2_PO_4_^−^ with the Tb.**1**(OTf)_3_ complex showed a concomitant increase in the π* to π transition and a decrease in the Tb-centered transition, indicating that the spectra tend towards one that resembles ligand **1**. The binding isotherm that was obtained by plotting the changes at the hypersensitive peak for Tb(III) emission, 545 nm versus equivalents of H_2_PO_4_^−^, is also shown. No discernable changes were seen in the shapes of the emission spectra from this titration indicating that perhaps the anion was not binding to the metal center or that the probe that was chosen was not one that could effectively monitor the interaction between anion and the first coordination sphere of the metal.

Shown in the bottom panel of Figure 4 is the series of absorption spectra obtained upon titration of [Tb.**1**](OTf)_3_ with H_2_PO_4_^−^. The disappearance of the shoulder at approximately 313 nm and a blue-shift of the peak at about 282 nm were observed—a spectrum at the end that resembled that of ligand **1**.

### 2.6. The Nature of the Sensor Response Towards Anions

Titration of anions resulted in quenching of the fluorescence of the [Tb.**1**](OTf)_3_ giving this Tb(III) complex a “turn-off” designation. This “turn-off” response, in our opinion, is due to the following:

(1) Binding of the anion leads to partial decomplexation of one or both terpyridine moieties from the metal center, thus inhibiting its ability to act as a sensitizer.
(2) Anion is bound to the sensitizer and or metal center in such a way that it “interferes” with the energy transfer from the triplet state of the ligand to the Tb excited state.

The anions displaced CH_3_CN molecules which had limited quenching ability in the first place. Allen and co-workers determined that the luminescence decay rate per inner sphere solvent molecule for Eu(III) complexes was 0 for acetonitrile compared to 0.83 s^−1^ for water and 0.41 s^−1^ for methanol [43]. Depending on the position in the course of the titration, either factor (1) or (2) or a combination thereof could be at play. These factors coupled with the limited quenching ability of CH_3_CN could explain the “turn-off” response.

### 2.7. How Does the Sensor Respond to Anions Other than Dihydrogen Phosphate?

**Figure 5:**
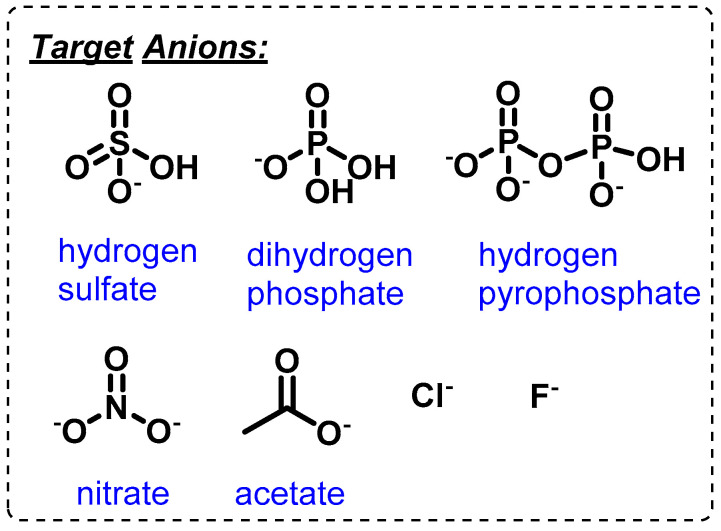
Anions targeted in this study.

To determine how the sensor would respond towards a variety of anions, we chose: HSO_4_^−^, NO_3_^−^, the phosphate derivatives (H_2_PO_4_^−^, HP_2_O_7_^3−^), the halides (Cl^−^ and F^−^) and the carboxylate, CH_3_CO_2_^−^—a diverse enough set of anions which allowed us to gain an understanding of how the sensor responds to factors such as charge, basicity, size, shape and the presence of rotamers that could lead to luminescence quenching (Figure 5).

**Figure 6:**
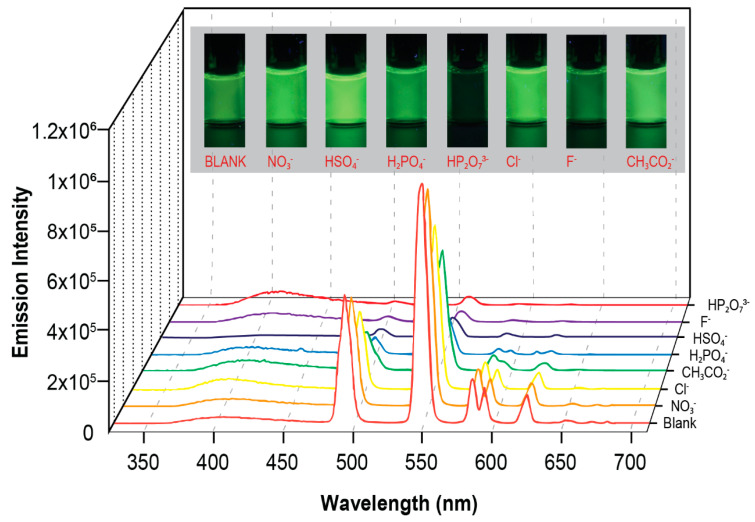
Steady state emission spectra of [Tb.**1**](OTf)_3_ (9.9 µM) in the presence of 1 equivalent of anions. Pictures show [Tb.**1**](OTf)_3_ (50 µM) in the presence of 1 equivalent of each anion.

**Figure 7:**
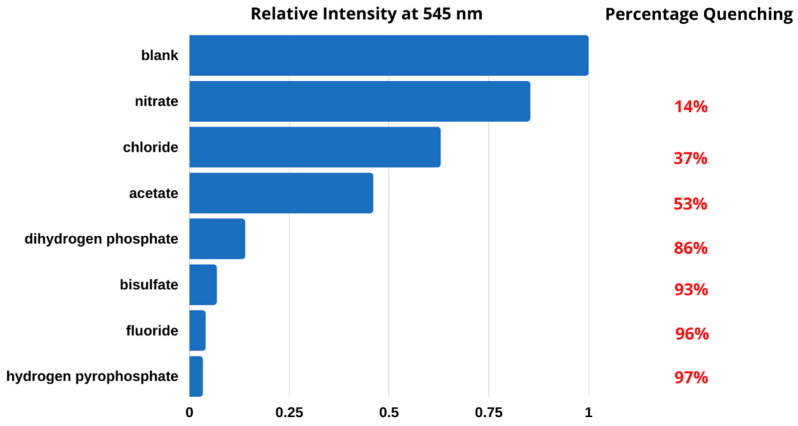
Relative intensity values at 545 nm and the corresponding percent quenching for Tb.**1**(OTf_3_) in the presence of 1 equivalent of anions.

Figure 6 shows steady state emission spectra representative of [Tb.**1**](OTf)_3_ (9.9 µM) in the presence of 1 equivalent of each anion. These spectra were then translated to relative intensity and percent quenching ability (at 545 nm) (Figure 7) by comparing the spectrum in the presence of each anion to a solution of the [Tb.**1**](OTf)_3_ in the absence of anion. One equivalent was chosen as the readout point as we felt that this point struck a balance between being far enough along in the titration where there was a fair amount of quenching yet, a differential sensor response towards anions could be observed. A series of vials representing the [Tb.**1**](OTf)_3_ (50 µM) in the presence of 1 equivalent of each anion when exposed to UV light is also shown in Figure 6.

Two key factors appear to be at work when one examines the overall sensor response: (1) the Lewis (and Bronsted) basicities of the anions [44,45,46] and (2) the number of OH bonds that the anion possesses. The anions HSO_4_^−^ and HP_2_O_7_^3−^ each contain one OH bond, whereas H_2_PO_4_^−^ contains two OH bonds. These OH bonds are high energy oscillators (3600 cm^−1^) with the ability to quench the luminescence of the lanthanide sensor via non-radiative decay. This decay is proportional to the number of O-H bonds present and the distance from the metal center [47].

The quenching ability runs parallel to the basicity of the anions, generally, with two exceptions. Anions HSO_4_^−^ and HP_2_O_7_^3−^ present with greater quenching ability that one would expect if basicity were the only factor at play. We assert that this greater quenching ability is due to an OH bond. The sensor response that was obtained is best considered in three cohorts. The weakest responses were obtained for the weakest bases (NO_3_^−^ and Cl^−^), good responses were obtained for CH_3_CO_2_^−^ and H_2_PO_4_^−^ and the best responses for F^−^, HSO_4_^−^ and HP_2_O_7_^3−^. The best responses are due to basicity (in the case of F^−^), “luminescent quenchable” OH bonds (HSO_4_^−^ and HP_2_O_7_^3−^) and the −3 charge (HP_2_O_7_^3−^).

**Figure 8:**
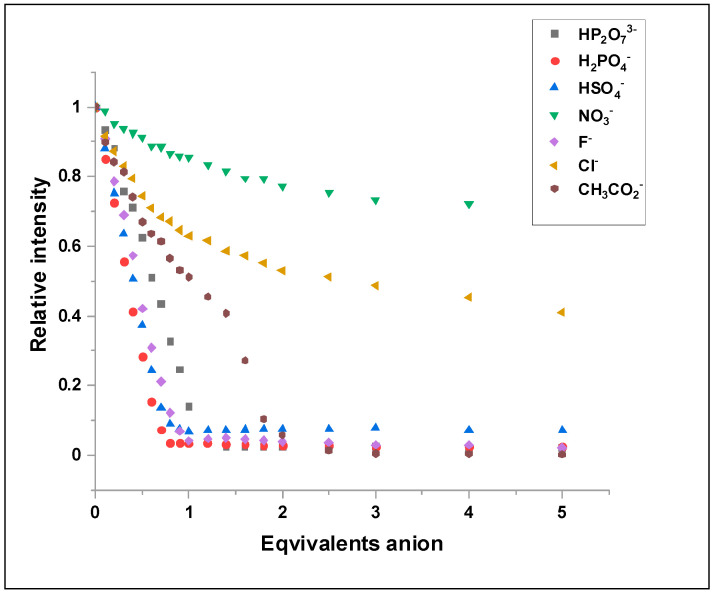
Binding isotherms showing the fluorescence intensity of [Tb.**1**](OTf)_3_ at 545 nm vs. equivalents of anion.

Quantitative information regarding the anion binding event was obtained from the titration of [Tb.**1**](OTf)_3_ (9.9 µM) with anions (0 to 5 equivalents). Binding isotherms (Figure 8) (normalized fluorescence intensity vs. anion concentration plots) were obtained by monitoring changes at the 545 nm peak analyzed using a 1:1 sensor: anion binding model in HypSpec. We, for comparison, show the binding isotherms with all anions at the same concentration but note that for some anions, higher (example: NO_3_^−^) and lower (example: HP_2_O_7_^3−^) concentrations were used in experiments so as to obtain the appropriate curvature, a necessity for accurate fitting of the isotherms to nonlinear regression models [48].

The binging constants of [Tb.**1**](OTf)_3_ with the anions NO_3_^−^, Cl^−^ and H_2_PO_4_^−^ were determined to be log *K* = 5.60 (±0.16), 6.23 (±0.33) and 6.50 (±0.33), respectively (See Figures S13–S15 in the Supplementary Information). Concerning HSO_4_^−^, F^−^, HP_2_O_7_^3−^ and CH_3_CO_2_^−^ anions, their log *K* values were higher than 7, and hence exceeded the value that can be reliably measured with the program. We note that for CH_3_CO_2_^−^, the fit was better to a 1:2 sensor: anion complex, but the log β also exceeded the value that can be reliably measured with the program. For this proof-of-principle study, the goals of which were to construct a lanthanide-based sensor, determine what types of anions that the sensor could respond to and the nature of the sensor response, the aim was to determine not the range but the lower limit of the binding constants of anions. These goals were all successfully achieved.

## 3. Experimental Section

### 3.1. Material and Instruments

NMR spectra were recorded on Bruker 400 and 500 MHz spectrophotometers (Bruker, Billerica, MA, USA). Chemicals shifts are referenced to the residual solvent peaks and given in parts per million (ppm) for ^1^H and ^13^C NMR Spectra. HRMS were acquired on an Agilent 6530 Q-TOF using electrospray ionization (Agilent, Santa Clara, CA, USA). UV-vis spectra were acquired on a Cary 100-Bio UV-vis spectrophotometer that was purchased from Agilent (Santa Clara, CA, USA) and a PerkinElmer Lambda 35 (PerkinElmer, Waltham, MA, USA). Fluorescence excitation and emission spectra were acquired on a Horiba FluoroMax-4 and a PTI-QM4 steady-state fluorimeter with a 75 W Xenon arc-lamp and a R928 photo multiplier tube. Both fluorimeters were purchased from HORIBA Scientific (Piscataway, NJ, USA). All pH measurements were taken with a Denver Instruments UltraBASIC electronic pH meter (Denver Instruments, Bohemiam, TX, USA). Chemicals were purchased from Sigma-Aldrich, Alfa Aesar or Strem Chemicals Inc. and, unless stated otherwise, were used as received. Deuterated solvents for NMR analysis were purchased from Cambridge Isotope Laboratories Inc. or Sigma-Aldrich. The following solvents were used for chromatography or other analysis and used as received: methanol GR ACS Special Anhydrous (EMD), Dichloromethane ≥ 99.9%, ChromAR^®^ for HPLC, for UV spectrophotometry (Macron) and Acetonitrile ≥ 99.8% for HPLC-GC (Sigma Aldrich). Solvents that were used in reactions were treated with a Vacuum Atmospheres Company Solvent Purification System (SPS) (Hawthorne, CA, USA) for drying prior to use or were of the special anhydrous kind and used as received.

### 3.2. Synthesis of Ligand **1** and Lanthanide Complexes

Ligand **1**. DIPEA (22.8 µL, 1.3 × 10^−4^ mol) was added to a suspension of compound **3** [6c] (50 mg, 6.6 × 10^−5^ mol), N-[2,2′;6′,2″]Terpyridin-4′-yl-propane-1,3-diamine [11] (40 mg, 1.3 × 10^−4^ mol), EDCI (25 mg, 1.3 × 10^−4^ mol) and HOBt (20 mg, 1.3 × 10^−4^ mol) in CH_2_Cl_2_ (30 mL). The reaction mixture was stirred under Ar until TLC analysis showed reaction completion (ca. 8 h). Reaction work-up was effected by diluting the reaction mixture to twice its volume and then washing with DI water and brine (3 times each). The CH_2_CL_2_ layer was then dried with anhydrous MgSO_4_.The solvent was removed in vacuo to yield a white residue which was purified via column chromatography (neutral alumina, 5% MeOH in CH_2_Cl_2_). The product was obtained as a white to cream-colored solid (43 mg, 48.2% yield).^1^H NMR (400 MHz, CD_3_CN, 300 K) δ (in ppm): 8.95 (t, *J* = 5.67 Hz, 2H), 8.57 (d, *J* = 4.56 Hz, 4H), 8.37 (broad s, 4H), 8.18 (s, 2H), 7.87 (t, *J* = 7.51Hz, 4H), 7.60 (broad s, 2H), 7.37 (m, 4H), 7.13 (s, 4H) 6.93 (s, 4H), 4.65 (s, 4H), 4.18–3.30 (dd, *J* = 13.9 Hz, *J* = 354.12 Hz, 8H), 3.68 (m, 4H), 3.43 (m, 4H), 2.07 (m, 4H), 1.09 (s, 18H), 1.06 (s, 18H) ^13^C NMR (214 MHz, CD_3_CN, 300 K) δ (in ppm): 169.44, 150.27, 150.06, 149.72, 144.2, 138.3, 138.7, 134.74, 128.31, 127.09, 126.41, 121.99, 75.72, 41.12, 37.45, 35.05, 34.54, 32.69, 32.04, 31.67, 31.37, 30.40, 30.10, 23.45, 14.44. HRMS (ESI-TOF) m/z: [M + 2H]^2+^ Calcd for C_84_H_95_N_10_O_6_ 670.37520; Found 670.37740. UV-vis (acetonitrile) λ_max_ [nm (× 10^4^ cm^−1^M^−1^)]: 282 (5.03), 254 (3.67) (sh). Fluorescence (acetonitrile) λ_max_ nm: 400.

[Eu.**1**](OTf)_3_: Ligand **1** (26.3 mg, 2.0 × 10^−5^ mol) and Eu(OTf)_3_ (11.8 mg, 2.0 × 10^−5^ mol) were added to a round bottom flask in 4 mL of dry CH_3_CN. The resulting solution was stirred at room temperature overnight under Ar. The solvent was removed in vacuo, and the resulting residue was dissolved in the minimum amount of CH_2_Cl_2_. The target complex was precipitated via the addition of hexanes as a cream-colored (20.0 mg isolated, 53%). HRMS (ESI-TOF) m/z: [M-H-3OTf^−^]^2+^ Calcd for C_84_H_93_EuN_10_O_6_ 744.32610; Found 744.32340. (UV-vis (acetonitrile) λmax [nm (× 10^4^ cm^−1^ M^−1^)]: 288 (4.0), 310 (3.0) (sh). Fluorescence (acetonitrile) nm: 407, 594, 614, 655, 691, 698.

[Tb.**1**](OTf)_3_: Ligand **1** (19 mg, 2.0 × 10^−5^ mol) and Tb(OTf)_3_ (8.6 mg) were added to a round bottom flask in 4 mL of dry CH_3_CN. The resulting solution was stirred at room temperature overnight under Ar. The solvent was removed in vacuo and the resulting residue was dissolved in the minimum amount of CH_2_Cl_2_. The target complex was precipitated via the addition of hexanes as a white solid (12.7 mg isolated, 46%), respectively. HRMS (ESI-TOF) m/z: [M-3OTf-H]^2+^ Calcd for C_84_H_93_N_10_O_6_Tb 748.32860; Found 748.32610. UV-vis (acetonitrile) λ_max_ [nm (× 10^4^ cm^−1^ M^−1^)]: 289 (1.5), 314 (01.0) (sh). Fluorescence (acetonitrile) nm: 489, 544, 580, 619, 648, 671, 678.

### 3.3. Metal Binding Studies

Protocol for UV-Vis and Fluorescence Studies: All solutions were prepared in dry degassed CH_3_CN. Solutions of Ligand **1** (9.7 µM) and Ln(OTf)_3_ (5.8 and 0.58 mM) were prepared. This was carried out so that 5 µL of a solution of Ln(OTf)_3_ delivered either 0.1 equivalent (0.58 mM) or 1 equivalent (5.8 mM) to the ligand during the titration. After each addition of Ln(OTf)_3_ to ligand **1**, the cuvette was inverted 2–3 times. After a three-minute equilibration period, the spectrum was taken.

### 3.4. Anion Binding Studies

Protocol for UV-vis and Fluorescence Studies: Anions (as their TBA salts), except TBAF, were dried under vacuum at 70 °C overnight prior to each titration. TBAF was dried under vacuum at 40 °C for about 4 h. Transferal of solvent for solution preparation was performed under an Ar atmosphere. All solutions were prepared in dry degassed CH_3_CN. Solutions of [Tb.**1**](OTf)_3_ (9.9 µM) and anions (as TBA salts) (6.0 mM and 0.6 mM) were prepared. This was carried out so that 2 µL of a solution of an anion delivered either 0.1 equivalent (0.6 mM) or 1 equivalent (6.6 mM) to [Tb.**1**](OTf)_3_ during the titration. After each addition of anion solution to [Tb.**1**](OTf)_3_, the cuvette was inverted 2–3 times. After a three-minute equilibration period, the spectrum was taken.

Protocol for Visual Detection Studies: All solutions were prepared in dry degassed CH_3_CN. A solution of [Tb.**1**](OTf)_3_ (500 µM) was prepared and added to vials. Solutions of anions (9 mM) were prepared. This was carried out so that 5 µL of a solution of an anion delivered 1 equivalent with respect to [Tb.**1**](OTf)_3_.

## 4. Conclusions

The development of lanthanide complexes based on a terpyridine-functionalized calix[4]arene ligand chelated with Eu(III) or Tb(III) has been reported. These complexes were synthesized in two steps and characterized using ^1^H NMR, ^13^C NMR and MS. The photophysical properties were also studied; high molar absorptivity values, modest quantum yields and luminescence lifetimes on the ms timescale were obtained. The anion sensing ability of the Tb complex was evaluated due to its higher quantum yield (in acetonitrile solution), and it was found via visual detection, UV-vis and fluorescence studies to be responsive towards a range of anions. Large binding constants (ranging from log K = 5.6 to >7) were obtained from fluorescence titrations and were indicative of strong binding between the sensor and anions. Developing a sensor array based on the diversification of this system is currently being investigated in our laboratory.

## Figures and Tables

**Table 1:** Photophysical properties of ligand **1** and lanthanide complexes in CH_3_CN at room temperature.

Species	Absorption	Emission		
	λmax (nm) (log ε) ^a^	λmax (nm)	Φ (%)	τ (ms)
Ligand **1**	282 (4.70)	394	b	b
[Tb.**1**]^3+^	289 (4.17), 314 (4.00)	543	4.5(0.2) ^c^ 4.7(0.2) ^d^	0.95(0.02) ^e^
[Eu.**1**]^3+^	288 (4.60), 310 (4.48)	613	0.24(0.05) ^c^ 0.26(0.04) ^d^	1.00(0.04) ^f^

^a^ log of the extinction coefficient, ^b^ not measured, ^c^ measured relative to cesium tris(6-carboxypyridine-2-carboxylato) lanthanide(III), ^d^ measured relative to tris(bipyridine)ruthenium(II) chloride, ^e 5^D_4_ → ^7^F_5_ transition, ^f 5^D_0_ → ^7^F_2_ transition.

## Data Availability

Data contained within the article and Supplementary Materials are available on request from the authors.

## Supplementary Materials

The following material is available online: Experimental details of quantum yield and lifetime measurements; NMR spectra (^1^H, ^13^C, COSY, HMQC, HMBC) of ligand **1;** UV-Vis and Fluorescence spectra of titrations of ligand **1** with Eu(OTf)_3_; UV-Vis and Fluorescence spectra of the titrations of [Tb.**1**](OTf)_3_ with anions and data fits from HypSpec 2014 models (PDF).

## Sample Availability

Samples of the compounds are not available from the authors.

## References

[1] Sessler J.L., Gale P., Cho W., Stoddart J.F., Rowan S.J., Aida T., Rowan A.E. (2006). Anion Receptor Chemistry.

[2] Begum R.A., Kang S.O., Day V.W., Bowman-James K. (2011). Structural Aspects of Anion Coordination Chemistry. Anion Coord. Chem..

[3] Kubik S. (2011). Receptors for Biologically Relevant Anions. Anion Coord. Chem..

[4] Gale P.A., Caltagirone C. (2011). Anion Sensors. Chemosensors.

[5] Wenzel M., Hiscock J.R., Gale P.A. (2012). Anion Receptor Chemistry: Highlights from 2010. Chem. Soc. Rev..

[6] Busschaert N., Caltagirone C., Van Rossom W., Gale P.A. (2015). Applications of Supramolecular Anion Recognition. Chem. Rev..

[7] Wang X., Chang H., Xie J., Zhao B., Liu B., Xu S., Pei W., Ren N., Huang L., Huang W. (2014). Recent Developments in Lanthanide-Based Luminescent Probes. Coord. Chem. Rev..

[8] Gunnlaugsson T., Pope S.J. (2014). Lanthanide Ion Complexes as Chemosensors. Luminescence of Lanthanide Ions in Coordination Compounds and Nanomaterials.

[9] Spangler C., Schäferling M., Hänninen P., Härmä H. (2010). Luminescent Chemical and Physical Sensors Based on Lanthanide Complexes. Lanthanide Luminescence Springer Series on Fluorescence (Methods and Applications).

[10] Lee M.H., Kim J.S., Sessler J.L. (2015). Small Molecule-Based Ratiometric Fluorescence Probes for Cations, Anions, and Biomolecules. Chem. Soc. Rev..

[11] Aletti A.B., Gillen D.M., Gunnlaugsson T. (2018). Luminescent/colorimetric Probes and (Chemo-) Sensors for Detecting Anions Based on Transition and Lanthanide Ion receptor/binding Complexes. Coord. Chem. Rev..

[12] Shinoda S., Tsukube H. (2011). Luminescent Lanthanide Complexes as Analytical Tools in Anion Sensing, pH Indication and Protein Recognition. Analyst.

[13] Hewitt S.H., Macey G., Mailhot R., Elsegood M.R.J., Duarte F., Kenwright A.M., Butler S.J. (2020). Tuning the Anion Binding Properties of Lanthanide Receptors to Discriminate Nucleoside Phosphates in a Sensing Array. Chem. Sci..

[14] Bünzli J.C.G., Eliseeva S.V., Hänninen P., Härmä H. (2010). Basics of Lanthanide Photophysics. Lanthanide Luminescence. Springer Series on Fluorescence (Methods and Applications).

[15] dos Santos C.M.G., Harte A.J., Quinn S.J., Gunnlaugsson T. (2008). Recent Developments in the Field of Supramolecular Lanthanide Luminescent Sensors and Self-Assemblies. Coord. Chem. Rev..

[16] Butler S.J., Parker D. (2013). Anion Binding in Water at Lanthanide Centres: From Structure and Selectivity to Signalling and Sensing. Chem. Soc. Rev..

[17] Shunmugam R., Tew G.N. (2008). Terpyridine-Lanthanide Complexes Respond to Fluorophosphate Containing Nerve Gas G-Agent Surrogates. Chem. Eur. J..

[18] Gupta K., Patra A.K. (2018). A Luminescent pH-Responsive Ternary Europium(III) Complex of β-Diketonates and Terpyridine Derivatives as Sensitizing Antennae—Photophysical Aspects, Anion Sensing, and Biological Interactions. Eur. J. Inorg. Chem..

[19] Ghosh S., Abbas Z., Dasari S., Patra A.K. (2017). Luminescent Eu^3+^ and Tb^3+^ complexes of 4-aminophenyl terpyridine (ptpy): Photophysical aspects, DNA and serum protein binding properties. J. Lumin.

[20] Hau F.K., Lo H., Yam V.W. (2016). Synthesis and Photophysical Studies of Calixarene-Based Alkynylplatinum(II) Terpyridine Complexes with various Receptor Sites for Colorimetric and Luminescence Sensing of Anions. Chem. Eur. J..

[21] Li L., Du L., Sun J., Yan C. (2013). Synthesis, Crystal Structure and Fluorescent Sensor Ability of Bis-Terpyridinyl-Calix[4]Arene Derivatives. Chem. Res. Chin. Univ..

[22] Molard Y., Parrot-Lopez H. (2002). Molecular Scaffolds for Di-Metallic Complexation: The Synthesis, Characterisation and Complexation Properties of Tetrakis-Terpyridinyl-Calix[4]Arene. Tetrahedron Lett..

[23] Muravev A.A., Agarkov A.S., Galieva F.B., Yakupov A.T., Bazanova O.B., Rizvanov I.K., Shokurov A.V., Zaitseva A.V., Selektor S.L., Solovieva S.E. (2020). New Terpyridine Derivatives of Thiacalix[4]Arenes in Solution and at the Water-Air Interface. Russ. Chem. Bull..

[24] Baldini L., Sansone F., Casnati A., Ungaro R. (2012). Calixarenes in Molecular Recognition. Supramolecular Chemistry.

[25] Kumar R., Sharma A., Singh H., Suating P., Kim H.S., Sunwoo K., Shim I., Gibb B.C., Kim J.S. (2019). Revisiting Fluorescent Calixarenes: From Molecular Sensors to Smart Materials. Chem. Rev..

[26] Jennings L.B., Shuvaev S., Fox M.A., Pal R., Parker D. (2018). Selective Signalling of Glyphosate in Water using Europium Luminescence. Dalton Trans..

[27] Caffrey D.F., Gunnlaugsson T. (2014). Displacement Assay Detection by a Dimeric Lanthanide Luminescent Ternary Tb(Iii)–cyclen Complex: High Selectivity for Phosphate and Nitrate Anions. Dalton Trans..

[28] Butler S.J. (2015). Quantitative Determination of Fluoride in Pure Water using Luminescent Europium Complexes. Chem. Commun..

[29] Shuvaev S., Starck M., Parker D. (2017). Responsive, Water-Soluble Europium(III) Luminescent Probes. Chem. Eur. J..

[30] Dickins R.S., Aime S., Batsanov A.S., Beeby A., Botta M., Bruce J.I., Howard J.A.K., Love C.S., Parker D., Peacock R.D. (2002). Structural, Luminescence, and NMR Studies of the Reversible Binding of Acetate, Lactate, Citrate, and Selected Amino Acids to Chiral Diaqua Ytterbium, Gadolinium, and Europium Complexes. J. Am. Chem. Soc..

[31] Edwards N.Y., Liu F., Chen G. (2013). Experimental and Computational Studies of Anion Recognition by Pyridine-Functionalized Calixarenes. Supramol. Chem..

[32] dos Santos C.M.G., Gunnlaugsson T. (2009). The Recognition of Anions using Delayed Lanthanide Luminescence: The use of Tb(Iii) Based Urea Functionalised Cyclen Complexes. Dalton Trans..

[33] dos Santos C.M.G., Fernandez P.B., Plush S.E., Leonard J.P., Gunnlaugsson T. (2007). Lanthanide Luminescent Anion Sensing: Evidence of Multiple Anion Recognition through Hydrogen Bonding and Metal Ion Coordination. Chem. Commun..

[34] Umali A.P., Anslyn E.V. (2010). A general approach to differential sensing using synthetic molecular receptors. Curr. Opin. Chem. Biol..

[35] Kotova O., Daly R., dos Santos C.M.G., Boese M., Kruger P.E., Boland J.J., Gunnlaugsson T. (2012). Europium-Directed Self-Assembly of a Luminescent Supramolecular Gel from a Tripodal Terpyridine-Based Ligand. Angew. Chem. Int. Ed..

[36] Gans P., Sabatini A., Vacca A. (1999). Determination of Equilibrium Constants from Spectrophotometric [Sic] Data obtained from Solutions of Known pH: The Program pHab. Ann. Chim..

[37] Marie C., Miguirditchian M., Guillaumont D., Tosseng A., Berthon C., Guilbaud P., Duvail M., Bisson J., Guillaneux D., Pipelier M. (2011). Complexation of Lanthanides(III), Americium(III), and Uranium(VI) with Bitopic N,O Ligands: An Experimental and Theoretical Study. Inorg. Chem..

[38] Canard G., Koeller S., Bernardinelli G., Piguet C. (2008). Effective Concentration as a Tool for Quantitatively Addressing Preorganization in Multicomponent Assemblies: Application to the Selective Complexation of Lanthanide Cations. J. Am. Chem. Soc..

[39] Montalti M., Prodi L., Zaccheroni N., Charbonniere L., Douce L., Ziessel R. (2001). A Luminescent Anion Sensor Based on a Europium Hybrid Complex. J. Am. Chem. Soc..

[40] Nakamoto K. (1960). Ultraviolet Spectra and Structures of 2,2′-Bipyridine and 2,2′,2″-Terpyridine in Aqueous Solution. J. Phys. Chem..

[41] Crosby G.A., Demas J.N. (1971). Measurement of Photoluminescence Quantum Yields. Review. J. Phys. Chem..

[42] Recording Fluorescence Quantum Yields. http://www.horiba.com/fileadmin/uploads/Scientific/Documents/Fluorescence/quantumyieldstrad.pdf.

[43] Dissanayake P., Mei Y., Allen M.J. (2011). Luminescence-Decay as an Easy-to-use Tool for the Study of Lanthanide-Containing Catalysts in Aqueous Solutions. ACS Catal..

[44] Bordwell pKa Table. https://organicchemistrydata.org/hansreich/resources/pka/#pka_dmso_compilation.

[45] Muckerman J.T., Skone J.H., Ning M., Wasada-Tsutsui Y. (2013). Toward the accurate calculation of pKa values in water and acetonitrile. Biochim. Biophys. Acta (BBA) Bioenerg..

[46] Oliveri I.P., Di Bella S. (2017). Lewis Basicity of Relevant Monoanions in a Non-Protogenic Organic Solvent using a Zinc(II) Schiff-Base Complex as a Reference Lewis Acid. Dalton Trans..

[47] Lo W., Wong W., Law G. (2016). Friend Or Foe? the Role of Solvents in Non-Triplet, Intraligand Charge Transfer Sensitization of Lanthanide(III) Luminescence. RSC Adv..

[48] Anslyn E.V., Dougherty D.A. (2006). Modern Physical Organic Chemistry.

