# Supplementary material for: Terpyridine-Functionalized Calixarenes: Synthesis, Characterization and Anion Sensing Applications
Source: Molecules. 2020 Dec 27;26(1):87. doi: 10.3390/molecules26010087 (PMC7795700; doi:10.3390/molecules26010087)
Supplement: Supplementary file 1 [file molecules-26-00087-s001.pdf]

Supporting Information *for*:

Terpyridine-Functionalized Calixarenes: Synthesis, Characterization and Anion Sensing Applications

Nicola Y. Edwards[1,*], David M. Schnable [1], Ioana R. Gearba [2], and Jenna L. Strubhar[1]

[1]Department of Chemistry and Biochemistry, Misericordia University, Dallas, PA 18612

[2]Department of Chemistry, The University of Texas at Austin, Austin, Texas 78712

* nedwards@misericordia.edu

Table of Contents:

NMR Spectra of Ligand **1** .... SI 2

Metal Binding Studies .... SI 5

Determination of Quantum Yields .... SI 8

Lifetime Measurements .... SI 9

Anion Binding Studies .... SI 11

NMR Spectra of Compound 1

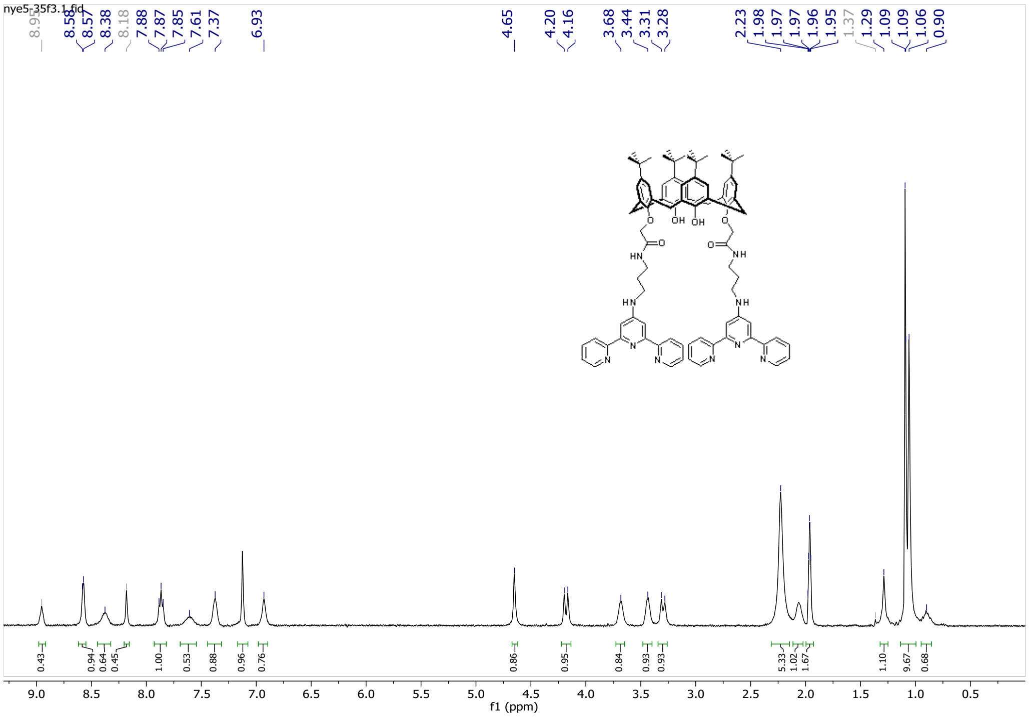

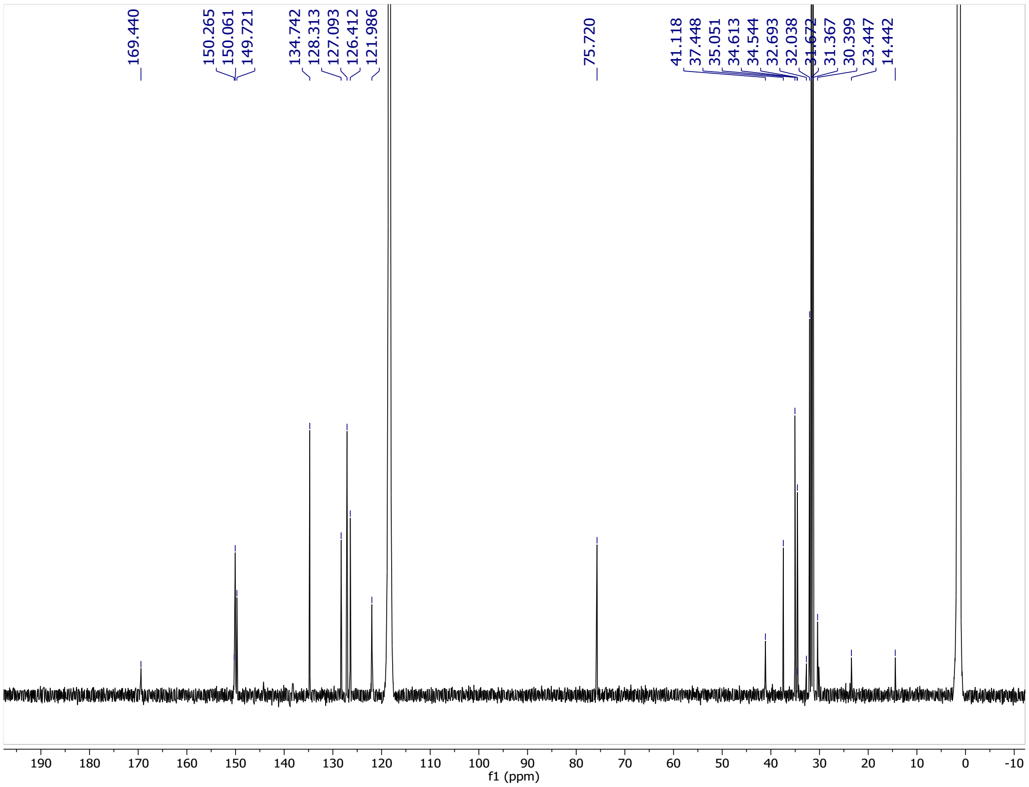

Figure S1. ^{1}H and ^{13}C NMR in CD_3CN of Compound 1

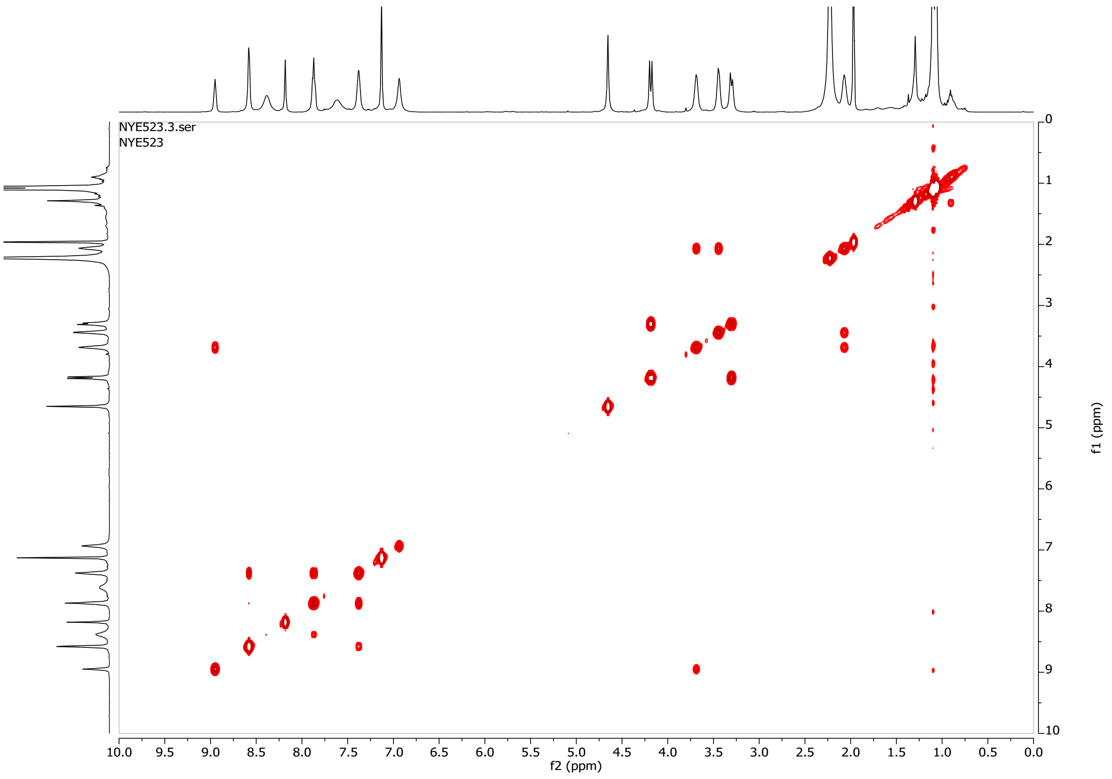

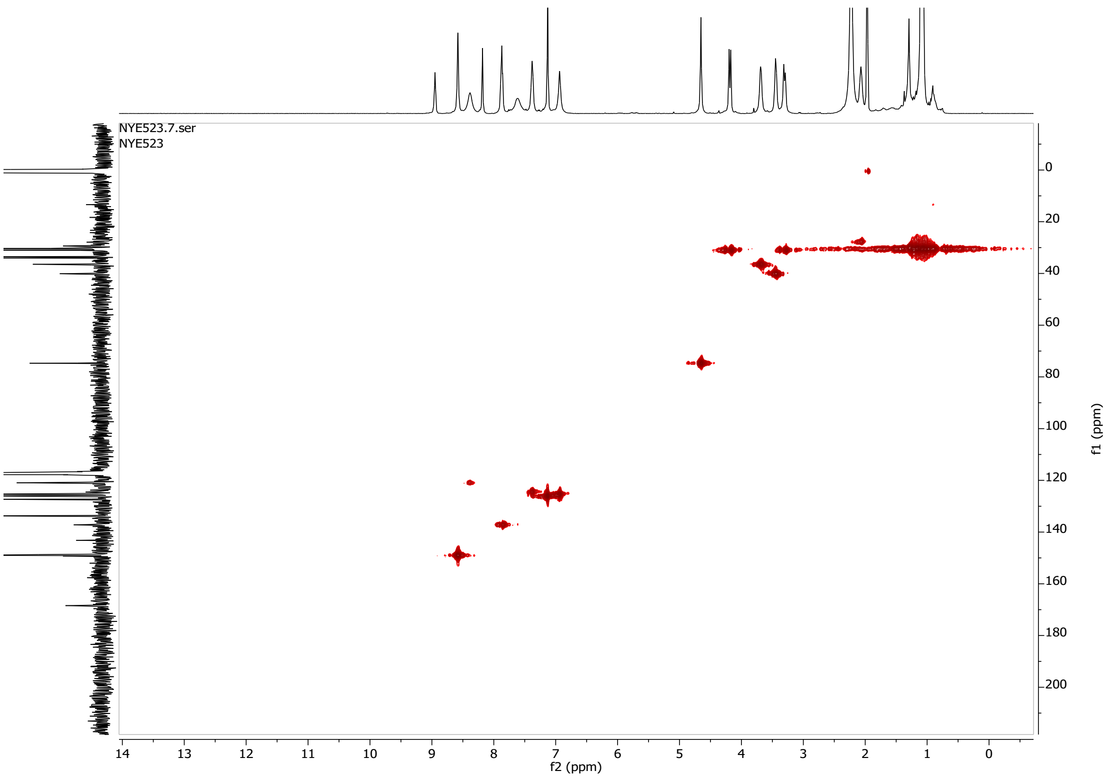

Figure S2. ^{1}H Cosy and HMQC in CD_3CN of Compound 1

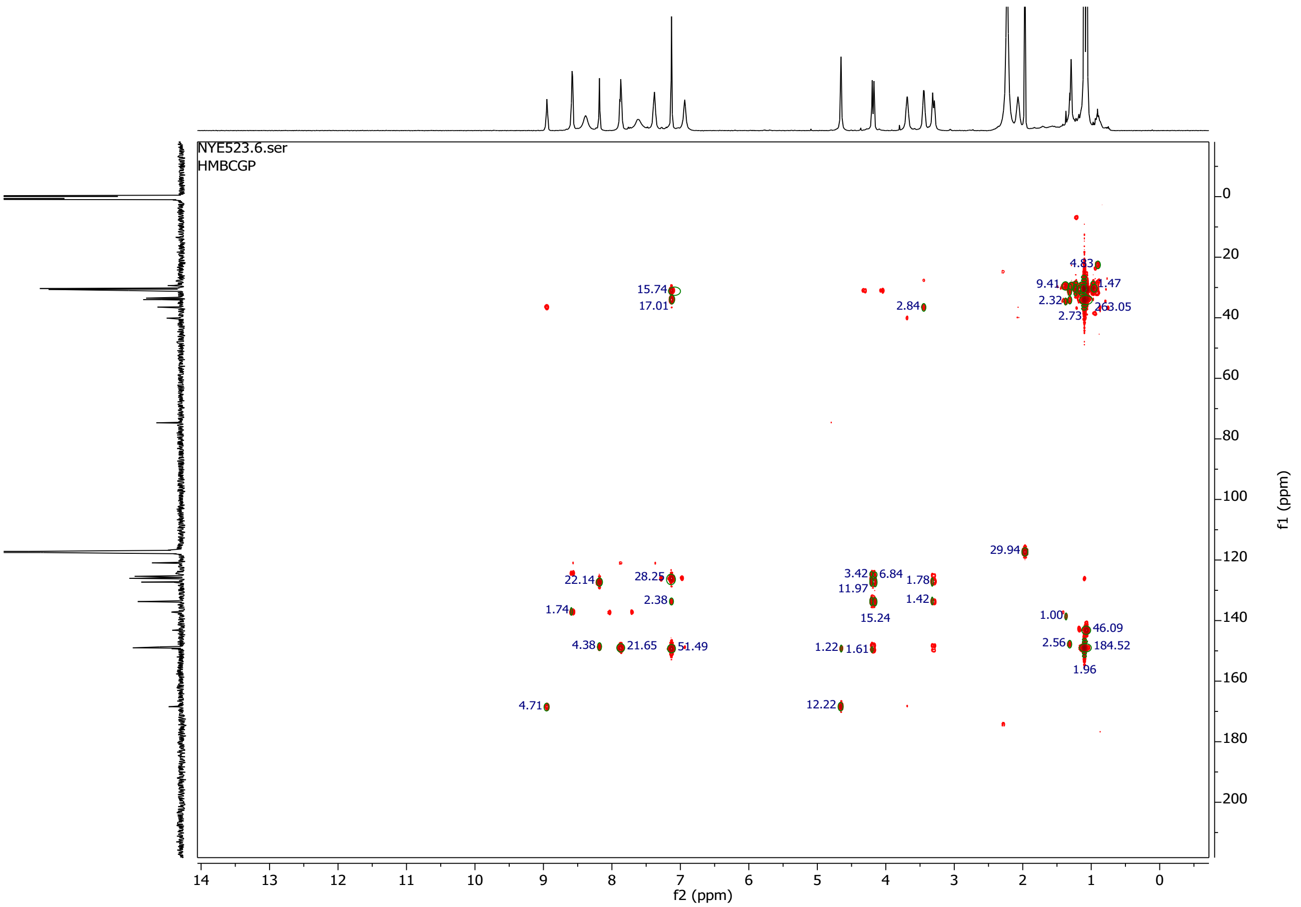

Figure S3. HMBC in CD_3CN of Compound 1

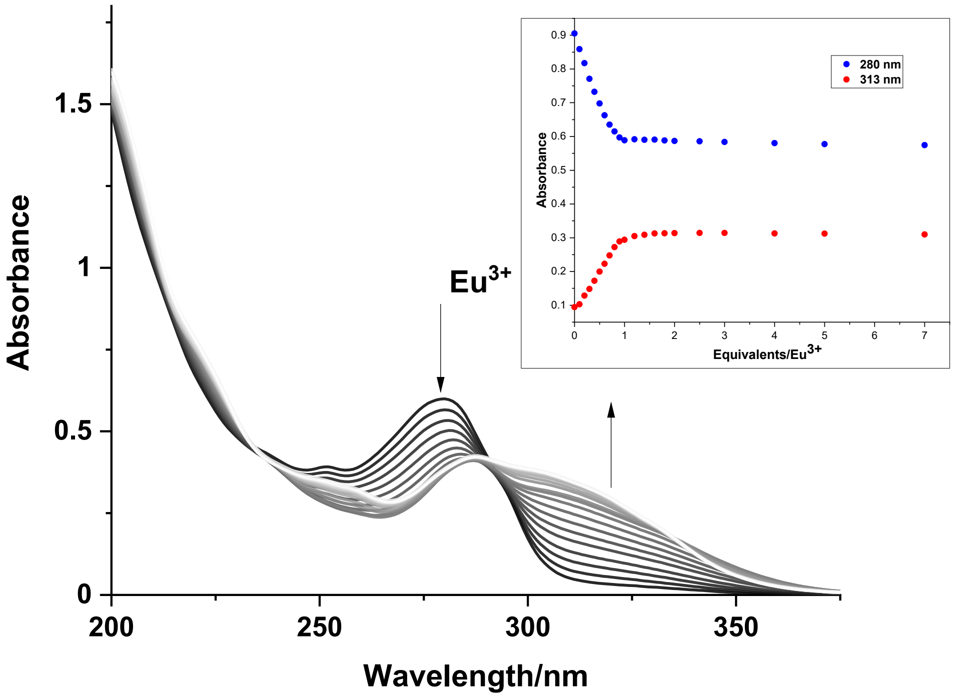

Figure S4. Absorption spectra representing the titration of ligand **1** (9.7 μM, acetonitrile) with $Eu(OTf)_3$. Inset: Absorbance vs. Equivalents Eu^{3+} at 280 nm and 313 nm.

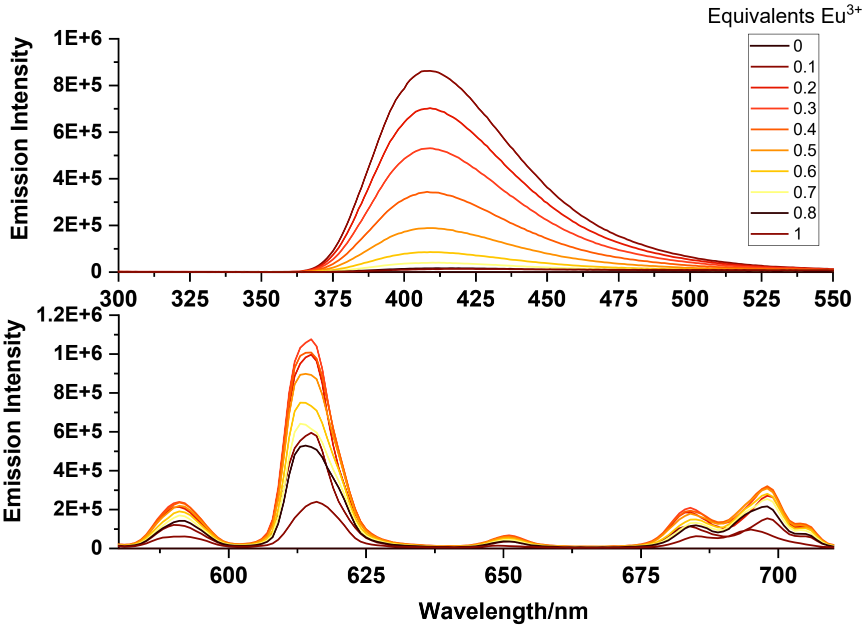

Figure S5. Fluorescence spectra representing the titration of ligand **1** (9.7 μM, acetonitrile) with 0 to 1 equivalents of $Eu(OTf)_3$. Excitation at 280 nm; excitation and emission slit widths were 3 nm. Integration time = 0.2s.

HYPSPEC 2014 Data		
Model is 1:1 ligand to metal		
Log β	value	standard deviation
AB	6.5966	0.0948

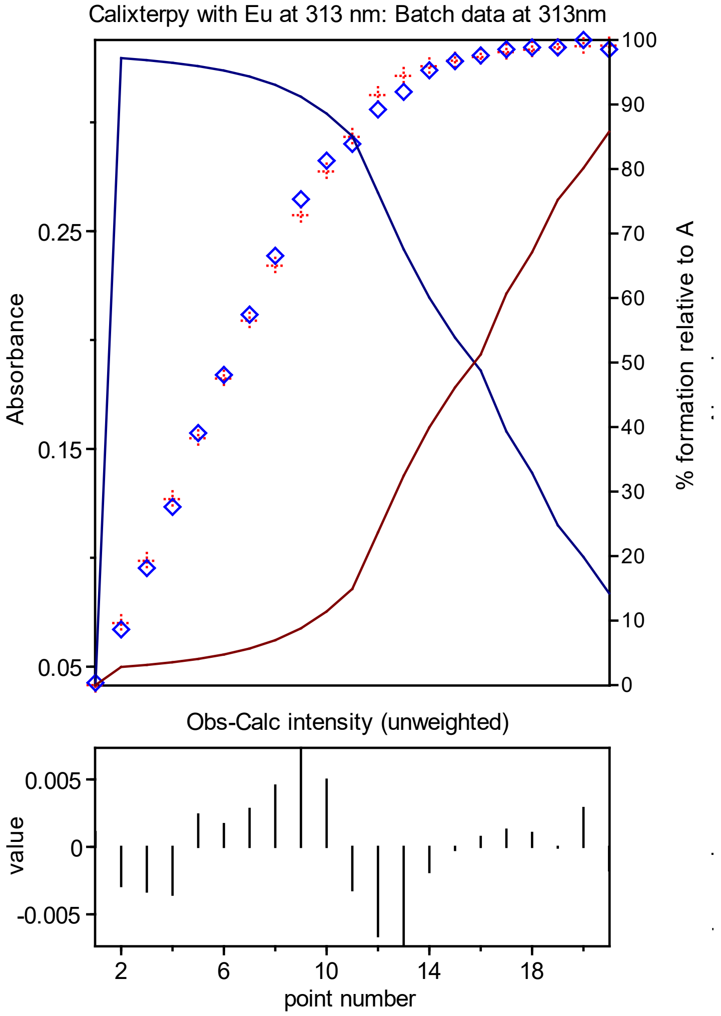

Figure S6. Observed and calculated intensities for the titration of ligand 1 with (9.7 μM, acetonitrile) with 0 to 7 equivalents of $Eu(OTf)_3$ at 313 nm.

HYPSPEC 2014 Data		
Model is 1:1 ligand to metal		
Log β	value	standard deviation
AB	7.2552	0.2432

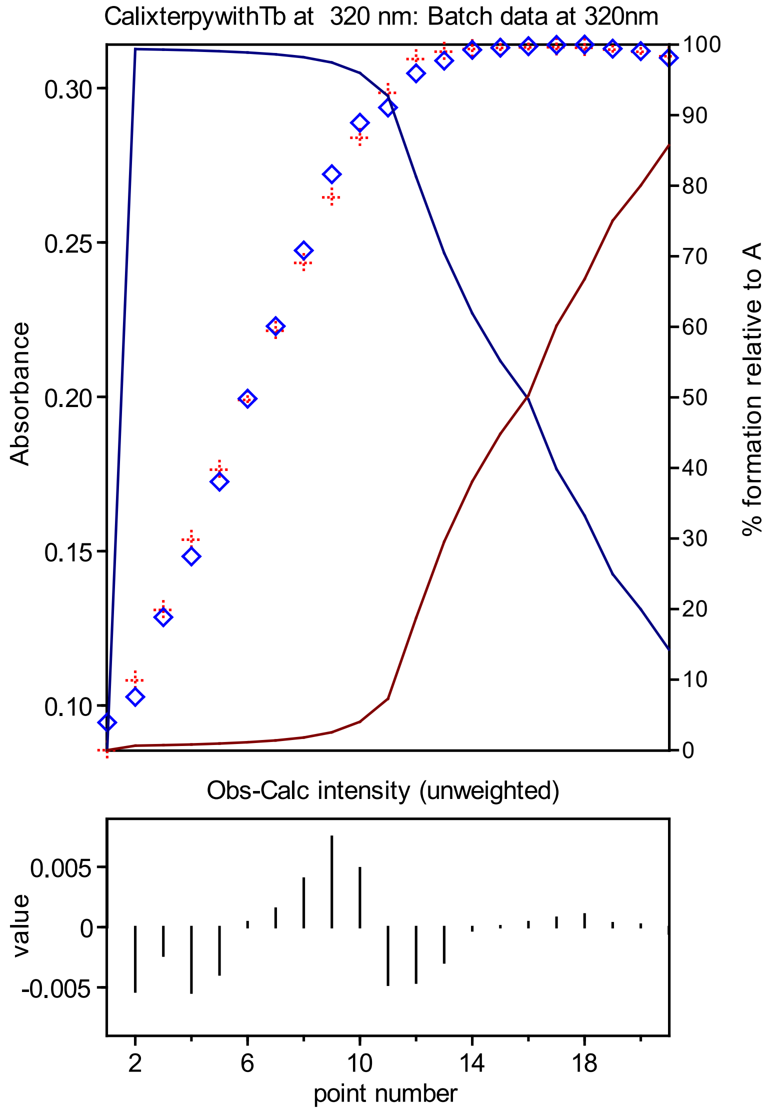

Figure S7. Observed and calculated intensities for the titration of ligand 1 with (9.7 µM, acetonitrile) with 0 to 7 equivalents of $Tb(OTf)_3$ at 320 nm.

Determination of Quantum Yields:

$Ru(bpy)_3Cl_3$ in degassed, neutral water (ϕ_f: 0.042) and the cesium dipicolato lanthanide(III) salts in 0.1M, pH 7.45 aqueous tris buffer ($\phi_{f, Tb}$: 22% ± 2.5%, $\phi_{f, Eu}$: 24% ± 2.5%) were used as standards [1]. Cesium Europium (III) and Terbium (III) dipicolinate salt standards were prepared according to the published procedure [2]. The compounds were dried under vacuum overnight before use. The standard $Ru(bpy)_3Cl_2$ was commercially available and was purchased from Sigma-Aldrich. Solutions of $Ru(bpy)_3Cl_2$ were prepared in degassed DI water. Solutions of both $Tb(dipic)^-$ and $Eu(dipic)^-$ were prepared in pH 7.45, 0.1 M tris buffer. Solutions of Tb.**1**.$(OTf)_3$ and Eu.**1**.$(OTf)_3$ were prepared dry degassed CH_3CN. Solution concentrations were chosen based on the literature molar extinction coefficient, such that the stock solution absorbance was *ca.* 0.1. These concentrations are as follows:

- $Ru(bpy)_3^{2+}$: 6.8 x10^{-6} M
- $Tb(dipic)^-$: 5.4 x 10^{-3} M
- $Eu(dipic)^-$: 5.4 x 10^{-3} M
- Tb.**1**.$(OTf)_3$: 1.5 x10^{-5} M
- Eu.**1**.$(OTf)_3$: 1.5 x10^{-5} M

To minimize the potential for inner filter effects, dilutions were performed yielding solutions of approximate absorbance values of 0.08, 0.06, 0.04, and 0.02. For all solutions, the UV-vis spectrum was recorded (220-600 nm), noting the absorbance at the excitation wavelengths:

- $Ru(bpy)_3^{2+}$: 286 nm
- Tb(dipic), Eu(dipic): 279 nm
- Tb.**1**.$(OTf)_3$ and Eu.**1**.$(OTf)_3$:289 nm

Subsequently, the full visible-range (330-700 nm) fluorescence spectrum was recorded using the noted excitation wavelength, below. The resultant fluorescence spectra were integrated across the emission range. The integration value was plotted against the absorbance of the solution at the excitation wavelength for all solutions. All solutions of each compound were plotted and fit to a linear trendline. The gradient of this trendline and reported standard error were used in order to calculate the quantum yield by the following:

$\phi_x = \phi_{st}\left(\frac{grad_x}{grad_{st}}\right)\left(\frac{\eta_x^2}{\eta_{st}^2}\right)$, whereas ϕ_x, η_x and $grad_x$ represent the fluorescence quantum yield, refractive index and gradient (slope) of the aforementioned linear trendline, respectively. Subscripts denote the identity of the solution, whether analyte ("x") or standard ("st").

The appropriate dipicolinate salt and $Ru(bpy)_3Cl_2$ were used as standards for compounds sharing the same metal center. The standards were first cross calibrated against one another, ensuring that they afforded a calculated quantum yield within 10% of the literature value for the standard. Following cross calibration, the gradients of the compounds were compared to each of the two standard compounds, individually. The quantum yields of the compounds were then calculated. The two values were checked to ensure that they were within 1%, then a simple average of the two

was taken to yield the experimentally obtained quantum yield. The absolute standard error in the slope of the gradient, as reported by the least squares linear fitting tool in the OriginLab suite of software, was converted to relative error and propagated through the quantum yield calculation to afford an error analysis given a single determination of quantum yield.

Lifetime Measurements:

Data were acquired using a Horiba Fluorolog 3 fluorimeter. Solutions of Tb.**1**.$(OTf)_3$ and Eu.**1**.$(OTf)_3$ with concentrations 10.3 µM and 18 µM respectively were prepared in dry and degassed CH_3CN. Solutions were placed in a quartz cell and loaded into the instrument. A Xe flash lamp (25 Hz repetition rate) with a spectral range of 230 to 600 nm and a 3 µs pulse width was used to excite the samples. The subsequent luminescence decay was probed at the most intense emission peak for each type of sample (615 nm for and Eu.**1**.$(OTf)_3$ and 545 nm for Tb.**1**. $(OTf)_3$).Two or three data sets were acquired for each sample. The data were analyzed using the data analysis software, DAS6 and in the OriginLab suite of software. The data were fit to a single exponential decay from which the rate constant and lifetime were extracted.

Measurement Parameters:

Excitation for Eu.**1**.$(OTf)_3$ and Tb.**1**.$(OTf)_3$: 280 nm, slit = 8 nm

Emission: 618 nm for Eu.**1**.$(OTf)_3$ and 546 nm for Tb.**1**.$(OTf)_3$, slit=6 nm

Preset: 5000 counts

Delay: 10%

Time gate: variable

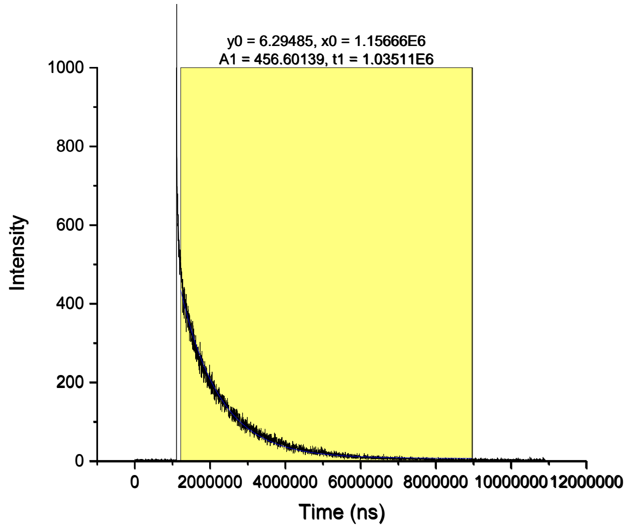

Figure S8. Example of Eu.**1**.$(OTf)_3$: luminescence decay and fit

Table S1: Lifetime measurements of Eu.**1**.$(OTf)_3$ and Tb.**1**.$(OTf)_3$ complexes

	Lifetime-Eu.**1**.$(OTf)_3$ (ms)	Lifetime-Tb.**1**.$(OTf)_3$ (ms)
**Run**	0.96	0.97
**Run**	0.96	0.97
**Run**	1.04	0.94
**Run**	1.05	0.93
**Average**	1.00	0.95
**Standard Deviation**	0.04	0.02

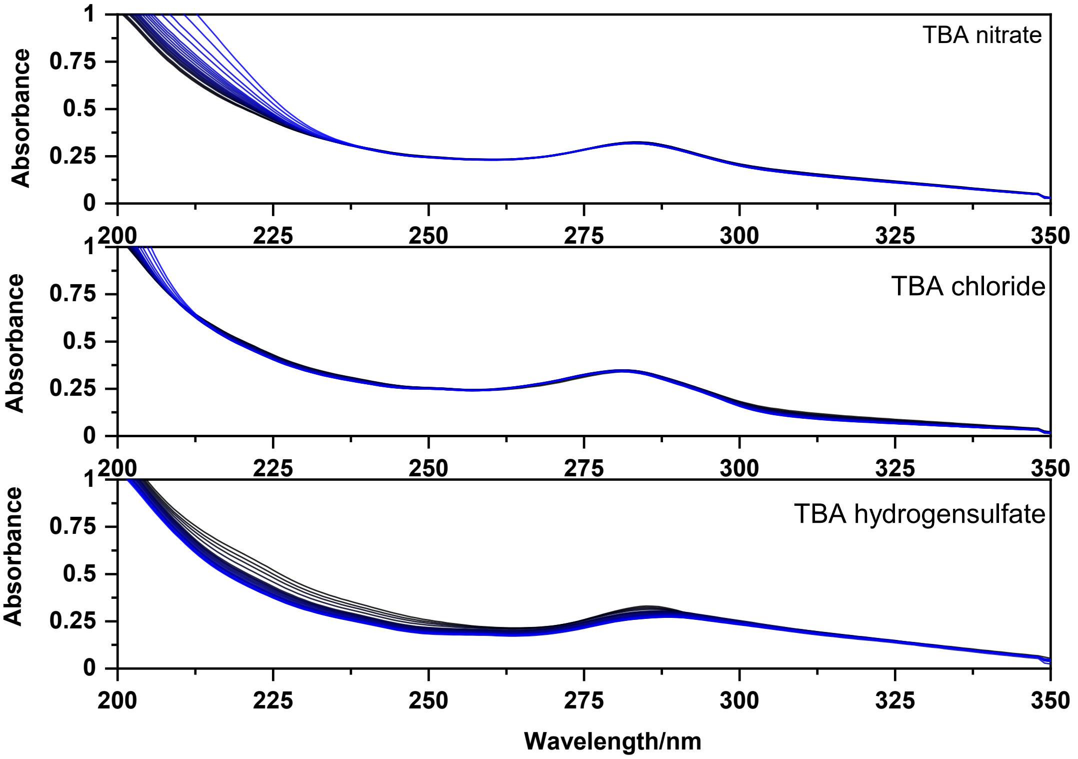

Figure S9. UV-vis spectra obtained from the titration of Tb.**1**.(OTf)$_3$ (9.9 µM) with 0 to 7 equivalents of various anions. Black spectra are at the beginning of the titration and blue spectra are at the end of the titration.

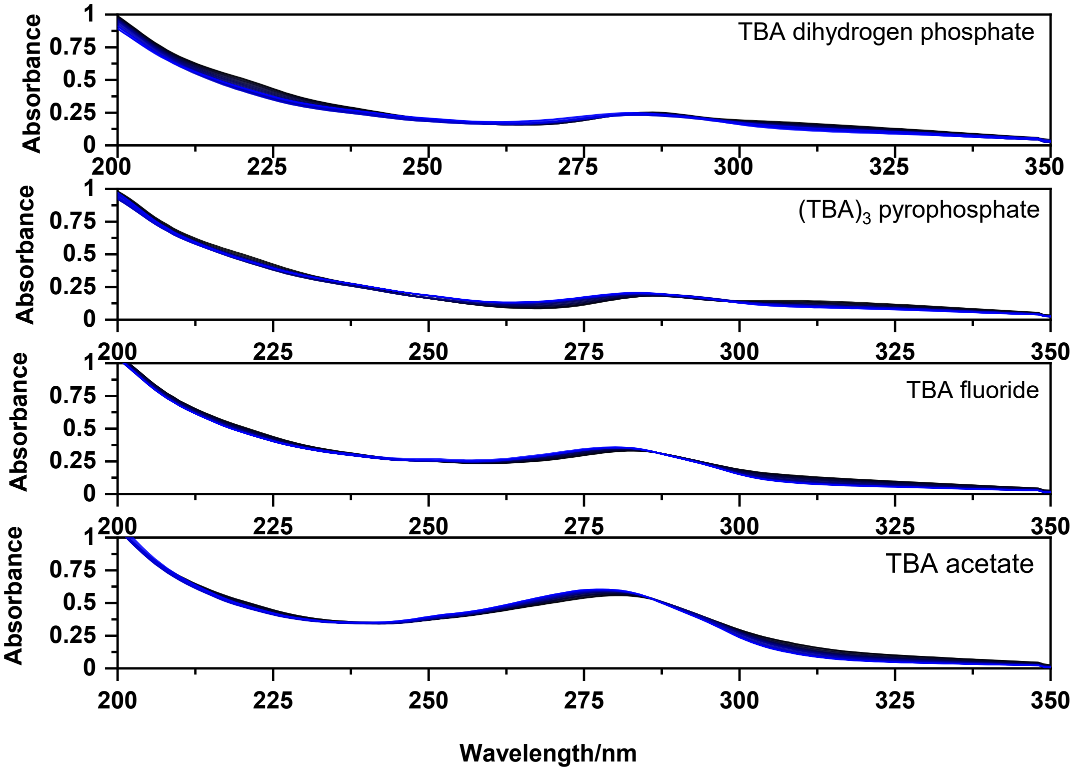

Figure S10. UV-vis spectra obtained from the titration of Tb.**1**.(OTf)$_3$ (9.9 μM) with 0 to 7 equivalents of various anions. Black spectra are at the beginning of the titration and blue spectra are at the end of the titration.

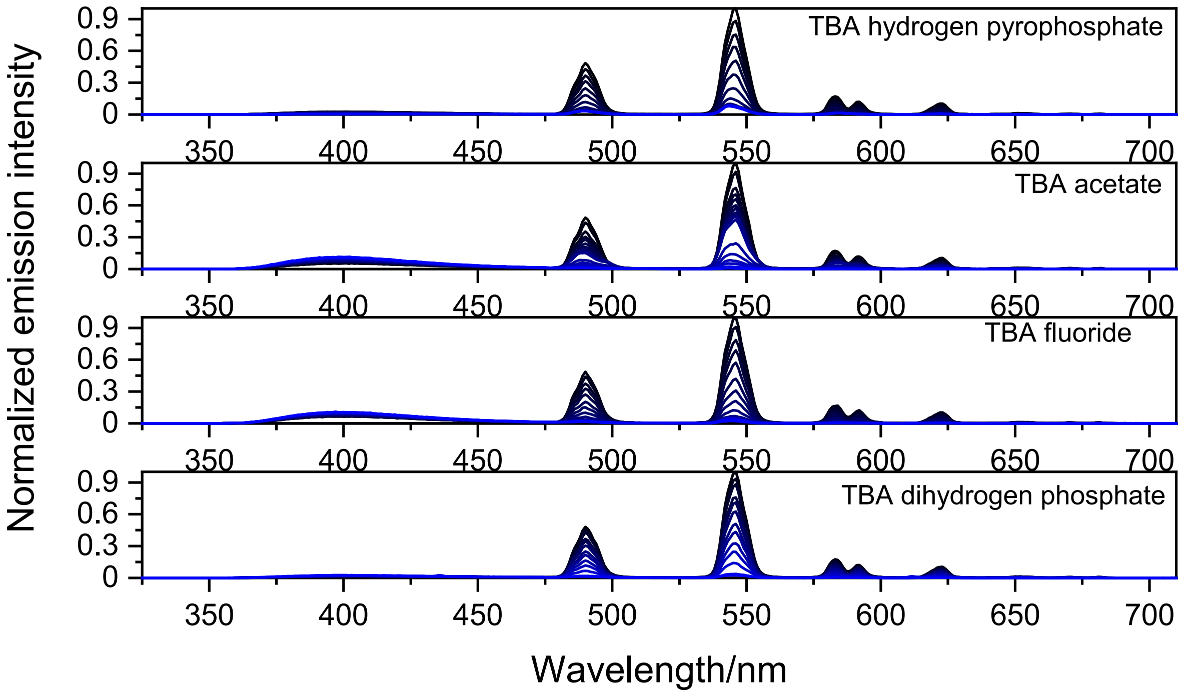

Figure S11. Fluorescence spectra obtained from the titration of Tb.**1**.$(OTf)_3$ (9.9 μM) with 0 to 7 equivalents of various anions. Black spectra are at the beginning of the titration and blue spectra are at the end of the titration.

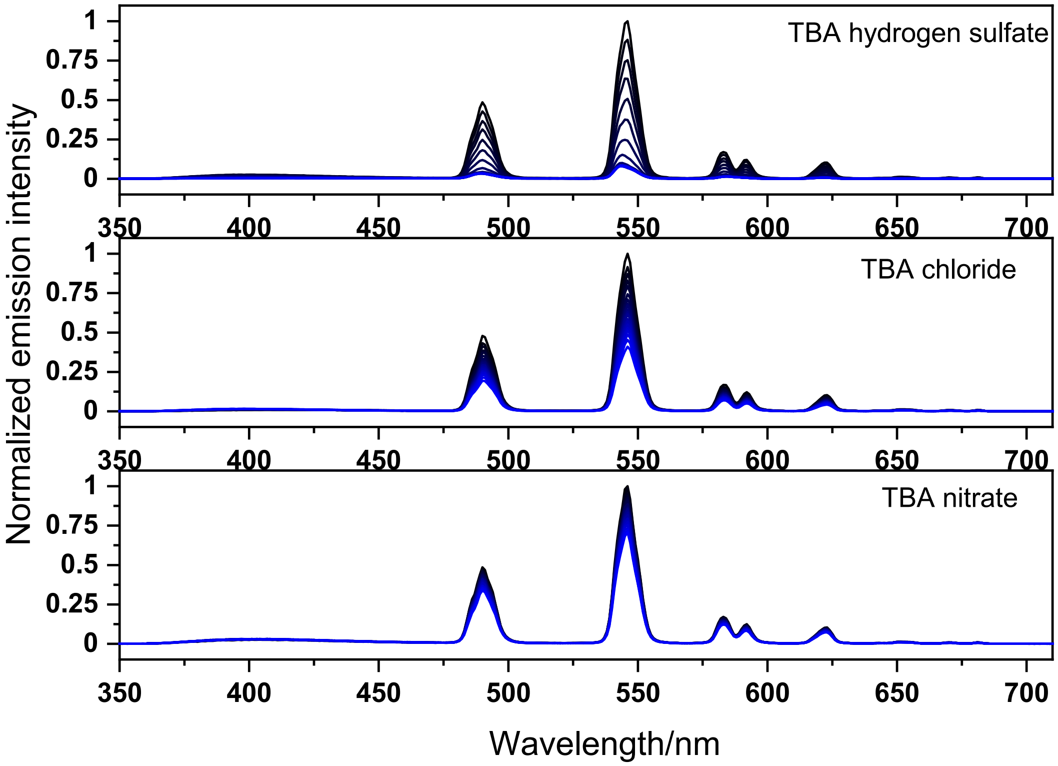

Figure S12. Fluorescence spectra obtained from the titration of Tb.**1**.$(OTf)_3$ (9.9 μM) with 0 to 7 equivalents of various anions. Black spectra are at the beginning of the titration and blue spectra are at the end of the titration.

Model is 1:1 ligand to metal		
Log β	value	standard deviation
AB	6.5013	0.3213

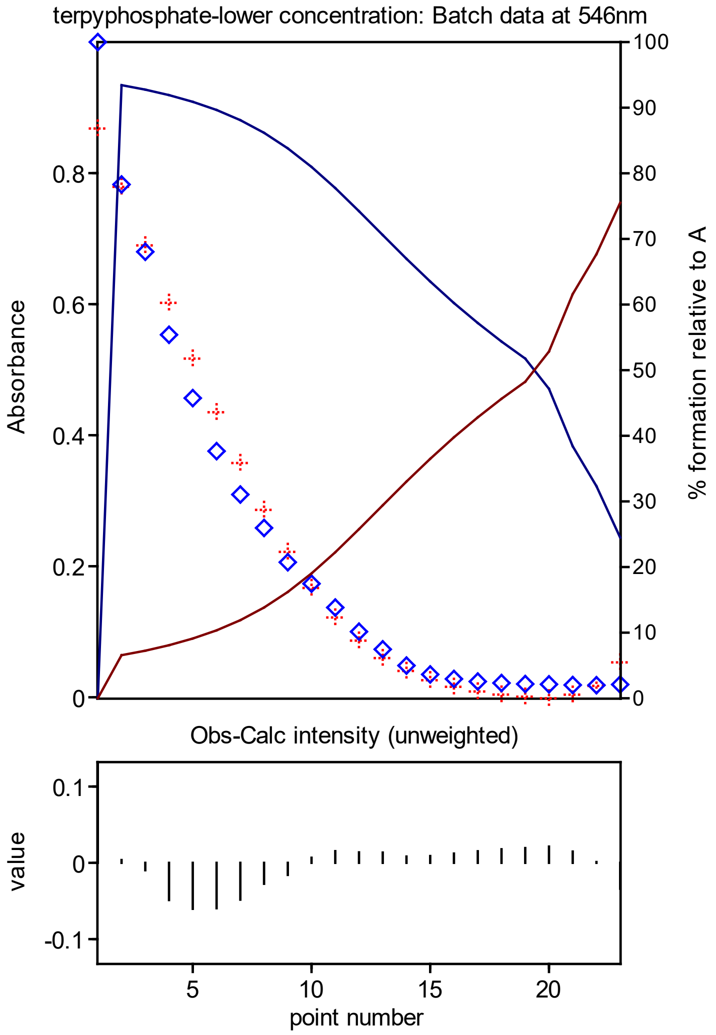

Figure S13: Observed and calculated intensities for the titration of Tb**1**.$(OTf)_3$ (4.95 µM, acetonitrile) with 0 to 5 equivalents of TBA dihydrogen phosphate at 546 nm.

Model is 1:1 ligand to metal		
Log β	value	standard deviation
AB	5.60	0.33

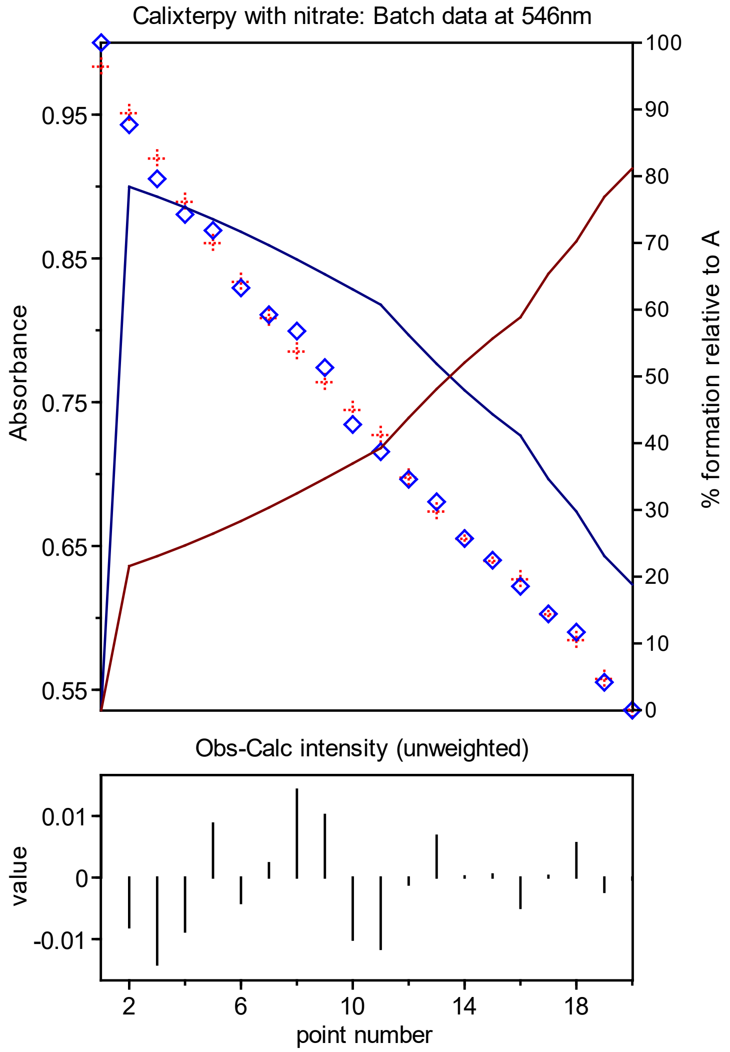

Figure S14: Observed and calculated intensities for the titration of Tb**1**.$(OTf)_3$ (9.9 μM, acetonitrile) with 0 to 5 equivalents of TBA nitrate.

Model is 1:1 ligand to metal		
Log β	value	standard deviation
AB	6.23	0.33

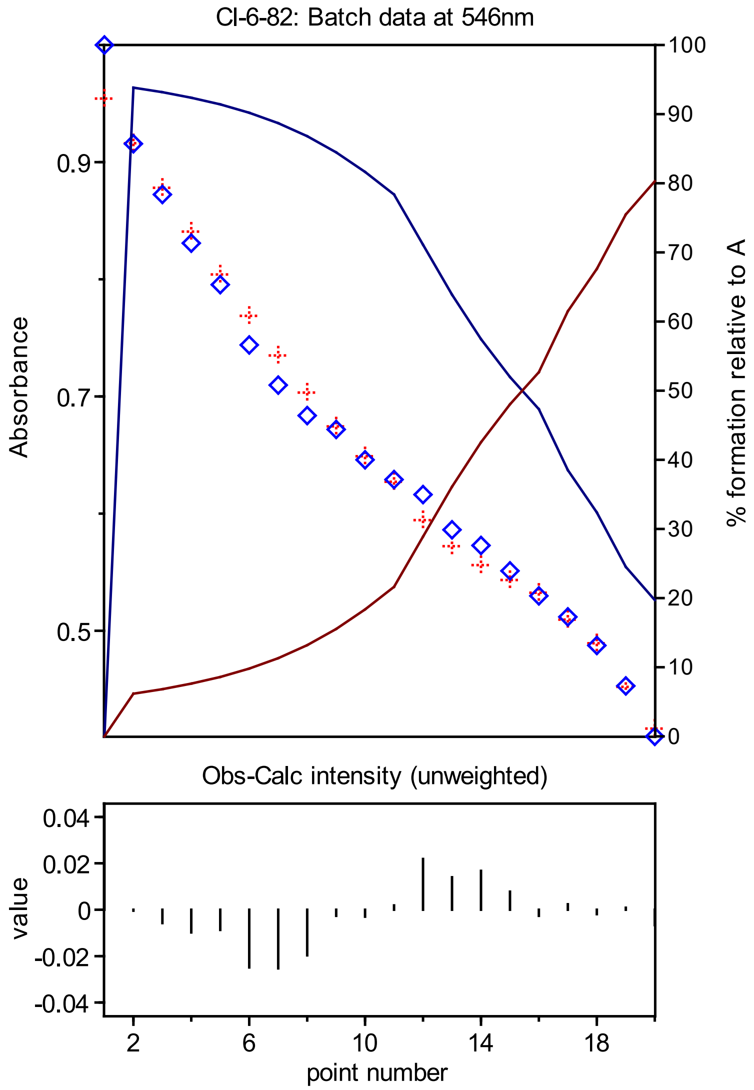

Figure S15: Observed and calculated intensities for the titration of Tb**1**.$(OTf)_3$ (9.9 µM, acetonitrile) with 0 to 5 equivalents of TBA chloride at 546 nm.

ABBREVIATIONS

Cesium Tris(6-carboxypyridine-2-carboxylato)terbium(III), $Tb(dipic)^-$; Cesium Tris(6-carboxypyridine-2-carboxylato)Europium(III), $Eu(dipic)^-$; tris(hydroxymethyl)aminomethane, Tris.

REFERENCES:

[1](a) Brouwer, A. M. Standards for Photoluminescence Quantum Yield Measurements in Solution (IUPAC Technical Report). *Pure and Applied Chemistry* **2011**, *83*, 2213-2228. (b) Chauvin, A.; Gumy, F.; Imbert, D.; Bünzli, J. G. Europium and Terbium Tris(Dipicolinates) as Secondary Standards for Quantum Yield Determination. **2004**, *37*, 517-532.

[2]Brayshaw, P. A.; Buenzli, J. G.; Froidevaux, P.; Harrowfield, J. M.; Kim, Y.; Sobolev, A. N. Synthetic, Structural, and Spectroscopic Studies on Solids Containing Tris(Dipicolinato) Rare Earth Anions and Transition Or Main Group Metal Cations. *Inorg. Chem.* **1995**, *34*, 2068-2076.
